# Super-wide-field two-photon imaging with a micro-optical device moving in post-objective space

**DOI:** 10.1038/s41467-018-06058-8

**Published:** 2018-09-03

**Authors:** Shin-Ichiro Terada, Kenta Kobayashi, Masamichi Ohkura, Junichi Nakai, Masanori Matsuzaki

**Affiliations:** 1 Department of Physiology, Graduate School of Medicine, The University of Tokyo, Tokyo, 113-0033 Japan; 2 Division of Brain Circuits, National Institute for Basic Biology, Aichi, 444-8585 Japan; 3 Graduate School of Biostudies, Kyoto University, Kyoto, 606-8501 Japan; 4 Section of Viral Vector Development, National Institute for Physiological Sciences, Aichi, 444-8585 Japan; 5 Brain and Body System Science Institute, Graduate School of Science and Engineering, Saitama University, Saitama, 338-8570 Japan; 6 Core Research for Evolutional Science and Technology, Japan Science and Technology Agency, Saitama, 332-0012 Japan; 7 International Research Center for Neurointelligence (WPI-IRCN), The University of Tokyo Institutes for Advanced Study, Tokyo, 113-0033 Japan

## Abstract

Wide-field imaging of neural activity at a cellular resolution is a current challenge in neuroscience. To address this issue, wide-field two-photon microscopy has been developed; however, the field size is limited by the objective size. Here, we develop a micro-opto-mechanical device that rotates within the post-objective space between the objective and brain tissue. Two-photon microscopy with this device enables sub-second sequential calcium imaging of left and right mouse sensory forelimb areas 6 mm apart. When imaging the rostral and caudal motor forelimb areas (RFA and CFA) 2 mm apart, we found high pairwise correlations in spontaneous activity between RFA and CFA neurons and between an RFA neuron and its putative axons in CFA. While mice performed a sound-triggered forelimb-movement task, the population activity between RFA and CFA covaried across trials, although the field-averaged activity was similar across trials. The micro-opto-mechanical device in the post-objective space provides a novel and flexible design to clarify the correlation structure between distant brain areas at subcellular and population levels.

## Abstract (web summary)

Recording the activity of neurons over large brain regions requires expanding the field of view of the optics without losing on spatial and temporal resolution. Here, the authors report a micro-opto-mechanical device that enables two-photon imaging across distant motor areas around 6 mm apart in the mouse.

## Introduction

Living organisms depend on large-scale cellular interactions. Inter-layer and inter-areal communications are especially crucial for information processing in the mammalian neocortex^1,2^. Even within the same cortical area, different layers receive synaptic inputs from different areas and send their output signals in different ways. Therefore, to understand the mechanisms of neocortical functions, it is essential to simultaneously measure the neural activity occurring at multiple layers in multiple areas. A potential way to address this issue in imaging of the mouse cerebral cortex is to expand the field of view (FOV) in two-photon laser scanning microscopy (TPLSM) because this microscopy technique allows detection of the activity of multiple neurons in all cortical layers and at a cellular resolution^3–8^.

In the common optical design of TPLSM, enlarging the FOV while maintaining the spatial resolution requires the diameter of the objective to be enlarged^9,10^. When the objective is redesigned, it is also necessary to redesign the pre-objective optics, ensuring that a satisfactory cellular resolution is attained; for the purposes of neuronal imaging, the axial resolution should be finer than 10–15 µm, which is the diameter of the neuronal soma^11^. Recently, two studies described development of ultra-large-sized objectives, with the FOV in TPLSM having been expanded to 3 mm with an axial resolution of 12 µm^12^, and 5 mm with an axial resolution of 4 µm^13^. These studies elegantly demonstrated that neural activity in multiple cortical areas within the large FOV (e.g., the primary and secondary visual cortices or the barrel and limb somatosensory cortices) could be simultaneously imaged at a cellular resolution. However, because of increasing optical aberrations towards the periphery of the FOV, the observable range for such methods is currently limited to 5 mm. An alternative method is to increase the number of FOVs, that is, the number of objectives. Lecoq et al.^11^ developed a dual-axis microscope possessing two individual scanning paths with two articulated arms that could simultaneously image two brain areas at an axial resolution of 10 µm. This was a versatile idea; however, each FOV was fixed by the mounting position of the objective. It is also difficult to image a large continuous area with this technique because there is a limit to how closely multiple objectives can be positioned while being made to face in the same direction.

In this study, we provide a novel concept to expand the FOV, which involves using the post-objective space in standard TPLSM; this does not require redesign of the objective and pre-objective space. So far, the post-objective space has attracted little attention because of the short working distances (WDs; <3 mm) of the high numerical aperture (NA) objectives used for two-photon imaging. Recently, high-NA objectives with a WD of up to 8 mm have been developed to detect fluorescence within the whole brain after tissue-clearing processing^14–16^. The present study describes the development of a micro-opto-mechanical device that rapidly rotates within the enlarged post-objective space. The device rapidly switches the FOV position, without movement of the objective or the sample. Combining this device with TPLSM enables sequential imaging of multiple distant (more than 6 mm) areas of the mouse brain, which can then be stitched together to form a large continuous image area (1.2 × 3.5 mm^2^) with a cellular resolution. We also demonstrate the utility of the device by studying functional correlations between two distant motor cortical areas at the levels of single axonal boutons, single neurons, and the neuronal population.

## Results

### Development of super-wide-field TPLSM

**Fig. 1:**
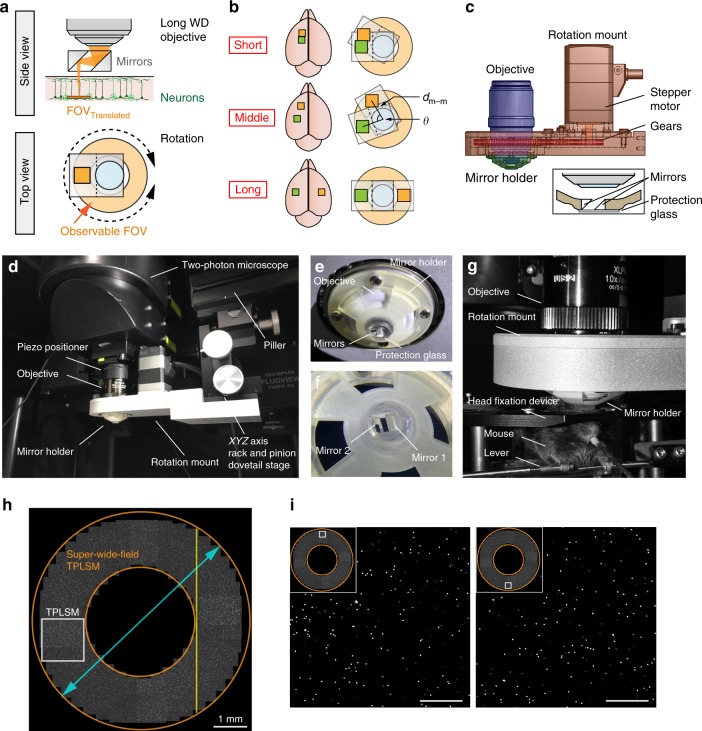
Design of the super-wide-field TPLSM. **a** Schema of the super-wide-field TPLSM. The excitation beam emitted from the objective is translated by a pair of mirrors. The orange donut shape (lower panel) represents the FOV observable by mirror rotation. **b** Left, schema for imaging of two distant areas in the mouse neocortex. Right, the distance between the two imaged areas (short, middle, and long) in the left image is determined by the inter-mirror distance (*d*_m–m_) and the rotation angle (*θ*). **c** Side view of the mirror holder with a pair of mirrors (green), a motorized rotation mount with three gears (red), and an objective (blue). The inset shows the median section of the mirror holder. **d** Photograph of the super-wide-field TPLSM. The holder with the mirrors is attached to the rotation mount and installed under a water-immersion objective connected to a two-photon microscope. The mount is supported by the pillar, independent of the microscope. The center position of the device is adjusted to fit with the optical axis of the objective using an *XYZ*-axis rack and pinion dovetail stage placed between the mount and the pillar. **e**, **f** Photograph of the holder with the mirrors. Mirrors 1 and 2 are the mirrors in the entering and exiting sides, respectively. **g** Photograph of the system for in vivo imaging of a behaving mouse. The mirror holder was set between the imaging window of the animal and the objective. The device occupied only the space above the head; therefore, there was sufficient space to place a head fixation device. **h** Measurement of the FOV observable by driving the micro-opto-mechanical device, and demonstrated by a stitched two-photon image of agarose-embedded fluorescent microbeads (2 µm). The white square represents a single FOV. The orange donut shape demonstrates the FOV observable by driving the micro-opto-mechanical device. The cyan arrow indicates the longest distance between the edges of the two observable FOVs. The yellow line indicates the longest line of stitch imaging. **i** Enlarged views of fluorescent microbeads located in two regions (regions are shown in the upper left insets) 6 mm apart. Scale bar, 100 µm

We devised a micro-opto-mechanical device that is placed under the objective of a standard upright TPLSM. The device consists of a pair of micromirrors, with their holder being attached to a mount and rotated perpendicularly to the optical axis of the objective (Fig. 1a). The mirror pair translates the optical pathway to another FOV. If the mirrors are rapidly rotated, the FOV can be rapidly switched. By rotating the mirror pair back and forth, the sample in two distant FOVs can be sequentially imaged (Fig. 1b). The distance between the centers of the two FOVs is *d*_m–m_ × 2 × sin (*θ*/2), where *d*_m–m_ is the distance between the mirrors and *θ* is the rotation angle. We used the micro-opto-mechanical device using an objective with a WD of 8.0 mm (XLPLN10XSVMP, ×10; Olympus; Fig. 1c–g and Supplementary Fig. 1). Each of the two mirrors had a height (*d*_mh_) of 2.5 mm, and *d*_m–m_ was set at 2.5 mm to secure the space above and below the device (Supplementary Fig. 2a). Therefore, the longest distance between the centers of the two FOVs was 5 mm at *θ* *=* 180°. To increase the amount of light passing through the mirrors and improve the spatial resolution, the width of the side of the mirror that was perpendicular to the optical path was set at 4 mm (Supplementary Fig. 2b–d). When the device was rotated at 10° intervals, fluorescent beads in a donut-shaped area were imaged as theoretically expected (Fig. 1h, i). The maximum observable distance was ~6.4 mm, which is 3.5-fold greater than the diameter of the original FOV, although the donut-shaped center area could not be imaged. The longest length of the line scan with stitch imaging was estimated to be ~5.2 mm (Fig. 1h). Therefore, we named this TPLSM with the micro-opto-mechanical device “super-wide-field TPLSM (super-wide-field TPLSM).”

**Fig. 2:**
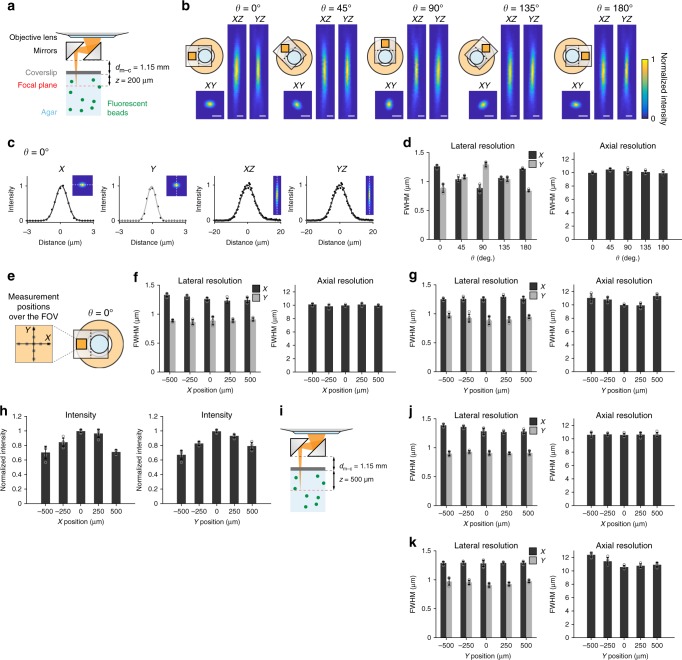
Optical performance of the super-wide-field TPLSM. **a** Measurement configuration for **a**–**h**. Agar-embedded 0.5 µm fluorescent microbeads were imaged through the device. **b**–**d** Lateral and axial FWHMs of the beads at the center of the FOV at five rotation angles. **b** Representative *XY*, *XZ*, and *YZ* images of the beads at five rotation angles. Scale bar, 2 µm. **c** Intensity profiles of the bead at *θ* of 0° (leftmost in **b**) along *X*-axis and *Y*-axis from the *XY* image, and *Z*-axis from the *XZ* and *YZ* images. The line profiles were acquired along the white dotted lines shown in the insets. Gray or black bold curves are fitted Gaussian curves. **d** Lateral and axial FWHMs at five angles. Each axial FWHM is an average of the axial FWHMs estimated from the *XZ* and *YZ* images. Five different beads were measured for each point. Gray dots indicate individual bead measurements. Data are plotted as mean ± s.d. **e** Measurement positions over the FOV for **f**–**h**, **j**, and **k**. *θ* was set at 0°. **f**, **g** Lateral and axial FWHMs of the beads along the *X*-axis (**f**) and *Y*-axis (**g**). **h** Normalized intensity of the beads along the *X*-axis and *Y*-axis. **i** The measurement configuration for **j** and **k**. **j**, **k** Lateral and axial FWHMs of the beads along the *X*-axis (**j**) and *Y*-axis (**k**)

To estimate the optical properties when imaging through the device, we measured the excitation point spread function of 0.5 µm fluorescent microbeads with two galvanometer scanners. The distance between the bottom surface of the mirror holder and the bottom surface of the coverslip for the glass window (*d*_m–c_) was set at 1.15 mm, and the distance between the bottom surface of the glass window and the focal plane, which corresponded to the imaging depth from the cortical surface in vivo (*z*), was 200 μm (Fig. 2a). With *θ* set to 0°, the full-width at half-maximum (FWHM) of the fluorescent microbeads at the center of the FOV was 1.26 ± 0.03 µm laterally for the *X*-axis direction, 0.88 ± 0.07 µm laterally for the *Y*-axis direction, and 9.96 ± 0.12 µm axially (mean ± s.d., *n* = 5; Fig. 2b–d). These values are comparable to those obtained in other studies using an ultra-large-sized objective and two gradient-index (GRIN) lenses^11,12^. The lateral FWHMs were slightly (about 0.4 µm or less) different between the *X*-axis and *Y*-axis, and depended on the rotation angle of the mirrors (Fig. 2d). This was consistent with the elliptic shape of the laser beam passing through the rectangular mirrors and the ratio of the major axis length to the minor axis length of the mirrors (4.0/2.5 = 1.6) (Supplementary Fig. 2d). However, the means of the lateral and axial FWHMs along the major and minor axes were similar across any angle (Fig. 2d). This result indicates that the rectangular mirror shape does not have large effects on the imaging of neuronal soma with a diameter of 10–15 µm. The FWHMs within a FOV showed degradation of ~20% from the center to the edge (500 µm from the center; Fig. 2e–g). The fluorescent intensity at the edge of the FOV was ~30% weaker than that at the center (Fig. 2h). These properties were similar when the mirrors were rotated at 45° (Supplementary Fig. 3).

In our design, *d*_m–c_ is constant during imaging because the mirror holder is supported independently of the objective (Fig. 1d). Therefore, when the focal plane is deepened, the distance between the objective exit and upper surface of the mirrors (*d*_o–m_) is shortened. This decreases the amount of light passing through the mirrors, and as a result the spatial resolution decreases. However, when *z* was set at 500 µm (*d*_m–c_ was set at 1.15 mm), the FWHMs of the microbeads at all axes over the FOV were similar to those at *z* = 200 µm (Fig. 2e–k). Furthermore, the degradation of the axial FWHM at the center of the FOV at a depth of 800 µm was only ~10% compared with that at a depth of 200 µm (Supplementary Fig. 4; *d*_m–c_ was set at 0.90 mm). With consideration of these results, we concluded that the similar spatial resolutions throughout the 1000 × 1000 µm^2^ area of the FOVs at a depth of up to *z* = 800 µm and at any rotation angle meant that calcium transients from single neuronal somata should be detectable throughout this imaging range, although the number of detected neurons at the periphery of the FOV might be smaller than that at the center.

**Fig. 3:**
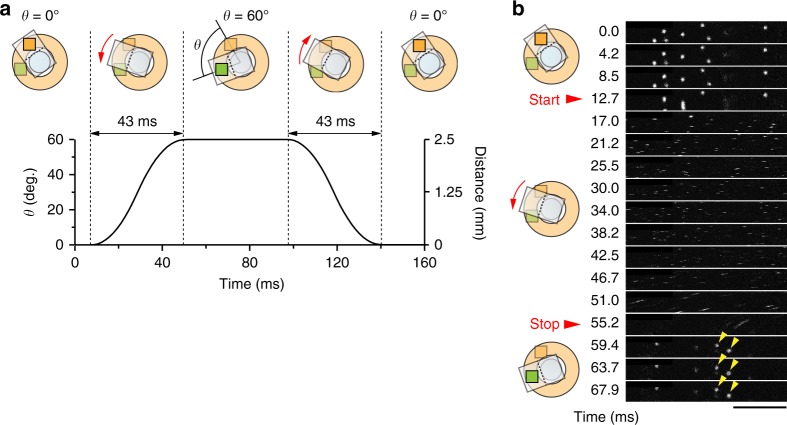
Mechanical performance of the super-wide-field TPLSM. **a** Representative time course of the rotation angle and the distance between the two FOVs. In this case, the FOV was moved from the orange field to the green field by rotating the device (represented by the rectangle) by 60°. The green field was then imaged before the device was moved in the opposite direction to image the orange field. Left, *Y*-axis, the true angle of the device (*θ*). Right, *Y*-axis, the distance between the centers of the two FOVs. **b** Time series of two-photon images of agarose-embedded fluorescent microbeads (2 µm) obtained by switching two FOVs at a distance of 2.5 mm. The frame rate was 236 frames s^−1^ and the FOV was 57 × 512 pixels. Scale bar, 40 µm

Next, we estimated the mechanical properties. Fast sequential imaging of multiple FOVs requires rapid rotation of the holder around the center of the first mirror (i.e., the optical axis of the objective). To perform this, we developed a mount equipped with a closed-loop stepper motor (Fig. 1c and Supplementary Fig. 1). It took 43 ms for the holder to rotate by 60° (Fig. 3a) and 80 ms for a 180° rotation. The true rotation angle of the device (*θ*) was measured by the internal position detector of the rotation motor. The distance between the centers of the two FOVs was then calculated as 2.5 mm × 2 × sin (*θ*/2). With an 8 kHz resonant scanner, the time to scan a FOV (512 × 512 pixels) is 33.3 ms; therefore, the ideal frame rate for dual-field imaging (*θ* = 60°, or 2.5 mm apart) is 6.6 Hz. To test the performance of the micro-opto-mechanical device, we continuously imaged fluorescent microbeads in two small FOVs at a distance of 2.5 mm during device rotation of 60° back and forth (Fig. 3b and Supplementary Movie 1). The timing of each device rotation was precise, with the image position being settled immediately after the end of the rotation (Fig. 3b).

### In vivo calcium imaging of dual or triple fields

**Fig. 4:**
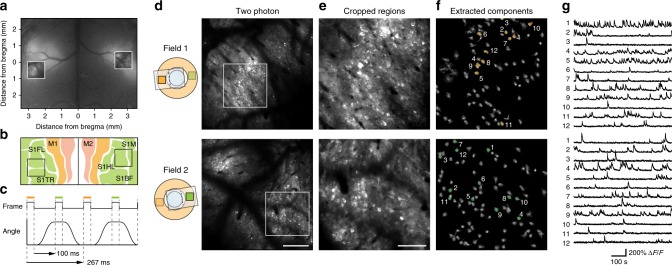
In vivo super-wide-field two-photon calcium imaging of bilateral S1. **a** An epifluorescence image of R-CaMP1.07-expressing neocortex. White squares indicate imaging fields in **d**. **b** A dorsal cortex map (modified from the Allen Brain Atlas^70^) and imaging fields in **d**. M1 primary motor area, M2 secondary motor area, S1M, primary somatosensory area (mouth), S1HL primary somatosensory area (hind limb), S1TR primary somatosensory area (trunk), S1BF primary somatosensory area (barrel field). **c** Timing chart of the dual-field imaging. Each field (orange, left S1; green, right S1) was imaged after changing the angle of the device. Dotted lines indicate the start and end timing of each frame acquisition. **d** Averaged two-photon images of layer 2/3 neuronal somata in the left S1 (top) and right S1 (bottom) in an awake mouse. The depth of the images was 150 μm from the cortical surface. Scale bar, 200 µm. **e** Enlarged views of the corresponding white squares in **d**. Scale bar, 100 µm. **f** Spatial components extracted by the constrained non-negative matrix factorization algorithm. **g** Representative Δ*F/F* traces of the 12 components numbered in **f** for each field. The raw fluorescence signals are shown to demonstrate the original imaging quality (see Methods for details)

To demonstrate that super-wide-field TPLSM can perform functional cellular imaging, we conducted sequential two-photon calcium imaging of two distant cortical areas, the left and right forelimb-related primary somatosensory cortices (S1FL). We prepared a mouse in which the red-shifted genetically encoded calcium indicator R-CaMP1.07^17^ was expressed in both areas (Fig. 4a, b). After fixing the mouse head under the microscope (see Methods for details), we rotated the device back and forth at *θ* *=* 177°, to image neuronal somata in layer 2/3 (L2/3) with a frame rate of 3.7 Hz (Fig. 4c–e and Supplementary Movie 2). The maximum distance of the two fields imaged was approximately 6.2 mm, which exceeded the maximum field size obtainable with the current state of TPLSM with a single objective (5 mm)^13^. From the acquired movie, neuronal somata with calcium transients were extracted using a constrained non-negative matrix factorization (CNMF) algorithm^18^ (Fig. 4f; see Methods for details). The identified neuronal somata showed clear calcium transients over the 7 min imaging period (Fig. 4g).

**Fig. 5:**
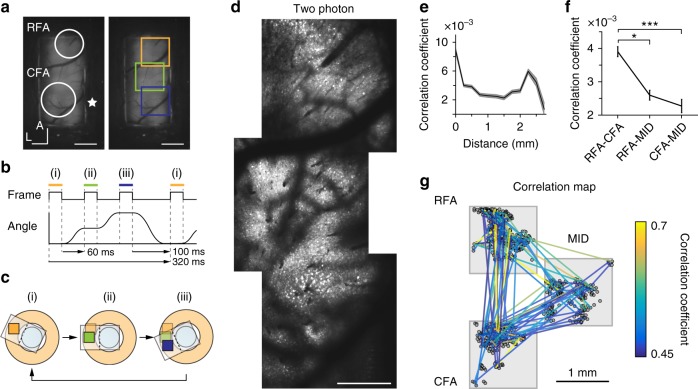
Stitch imaging of mouse motor cortex. **a** An epifluorescence image of left R-CaMP1.07-expressing motor cortex. The three squares indicate the imaging fields (orange, RFA; green, the intermediate region (MID); blue, CFA) in **d**. Scale bar, 1 mm. **b** Timing chart of the stitch imaging of the three fields. **c** Schematic illustration of stitch imaging. **d** Averaged two-photon images of the three areas shown in **a**. Scale bar, 500 µm. **e** Pairwise activity correlation as a function of the distance between the two neurons (see more details in Methods). Data were grouped into 250 µm bins. The black line represents the mean and the gray shading represents s.e.m. **f** Mean inter-areal correlations between pairs of neurons from RFA, CFA, and MID. RFA, MID, and CFA were defined as 1.5–2.5, 0.3–1.5, and −0.5 to 0.3 mm, respectively, from the anterior–posterior axis. *p < 0.05, ***p < 0.001, *n* = 93666 pairs in RFA-CFA, 87636 in RFA-MID, and 50794 in CFA-MID, Wilcoxon's rank-sum test. **g** Cell-to-cell correlation map. Small circles indicate the center of the region of interest. To clearly show long-range correlations, the regions of interest in MID are depicted 1 mm to the right. Lines connect pairs with a pairwise correlation >0.45. The correlation strength is pseudocolor coded

In addition to distant cortical areas, it is important to image a continuous wide field that contains adjacent cortical areas. By stitching between three adjacent FOVs, we imaged a 1.2 × 3.5 mm^2^ continuous L2/3 area at a frame rate of 3.1 Hz (Fig. 5a–d and Supplementary Movie 3); this area included two left forelimb motor areas, the rostral forelimb area (RFA) and caudal forelimb area (CFA)^19–21^. The three FOVs were smoothly connected, which indicates the planarity of the device rotation. Deconvolved calcium transients estimated from the CNMF algorithm (referred to as “inferred spike events”; see Methods for details) were filtered and used to calculate pairwise correlations. Pairwise correlation coefficients (CCs) between pairs of neurons <1 mm apart were negatively associated with the cellular distance (Fig. 5e), as previously reported for the mouse motor cortex^19,22^. In addition, a second peak in correlation appeared at a distance of about 2.3 mm, which corresponds to the distance between the centers of the RFA and CFA. These pairings between the RFA and CFA consistently showed a stronger correlation structure than pairings that included a neuron from the intermediate field (Fig. 5f, g). Thus, stitch imaging is a powerful tool that can identify distant but functionally related modules over the broad cortex at a cellular resolution. In the following studies, we focus on the neuronal activity in the RFA and CFA as functionally related modules.

### Identification of projecting axons with dual-field imaging

The great advantages of two-photon calcium imaging are the ability to measure the activity from subcellular structures such as dendrites^23,24^ and axons^25,26^, and the ability to measure cellular activity from deep cortical layers^5–8^. By narrowing the laser scanning angle to obtain higher-magnification images, we were able to sequentially image dendritic and/or axonal activity in layer 1 (L1) of the RFA and CFA (Supplementary Fig. 5). Imaging of neuronal somata at a depth of up to 800 µm from the cortical surface was also possible (Supplementary Fig. 6).

**Fig. 6:**
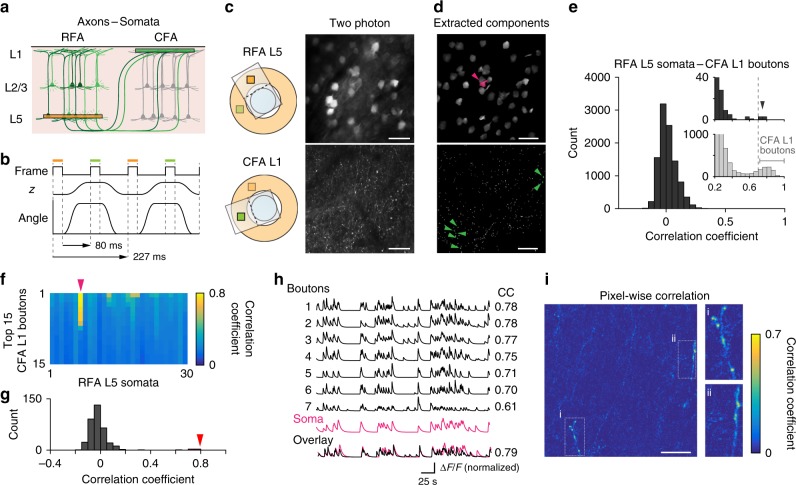
Dual-field imaging of neuronal somata and their projecting axons. **a** Schematic illustration of imaging fields (green, CFA; orange, RFA). **b** Timing chart during four consecutive imaging frames. **c** Left, schematic illustration of the FOV and device position at each imaging frame. Right, averaged two-photon images of GCaMP6s-expressing neuronal somata and axons. The depths of the fields in RFA L5 and CFA L1 were 450 and 25 µm, respectively. Scale bar, 50 µm. **d** All spatial components were extracted using the CNMF algorithm. Each component properly captured the structure of the neuronal soma and axonal bouton. Scale bar, 50 µm. **e** Histogram of pairwise correlation coefficients (CCs) in inferred spike events between the RFA L5 somata and CFA L1 boutons with >10 spike events. The upper right inset is an enlarged view of the histogram. The right middle inset is a histogram of CCs between CFA L1 boutons (gray bars). **f** CFA boutons with the top 15 CCs against each RFA soma are sorted in descending order of the CC values. A magenta arrowhead indicates the same neuronal soma as in **d**. **g** Histogram of CCs of the RFA L5 somata indicated by the magenta arrowhead in **d** and **f** against all CFA L1 boutons. A red arrowhead indicates the position of the top CCs (0.78). The standard deviation of the distribution was 0.12. **h** Representative Δ*F/F* (temporal components) traces of seven CFA boutons (black) that showed high CCs against the trace of the neuronal soma shown in **g** (magenta). The amplitude was normalized to the maximum Δ*F/F*. Positions of the soma and boutons are indicated in **d**. The number to the right of each trace is the correlation coefficient (CC) with the soma. **i** Left, map of pixel-wise correlations between raw fluorescent traces in the CFA L1 imaging field and temporal component traces of the RFA L5 neuronal soma indicated in **d**. To improve the signal-to-noise ratio, 5-frame moving averages of the raw fluorescent traces in the CFA L1 field were used. Right, enlarged maps of the two boxes shown in the left panel. Scale bar, 50 µm

Our previous anatomical study showed that a subset of layer 5 (L5) neurons in the RFA project to L1 in the CFA^27^. If RFA L5 and CFA L1 were sequentially imaged with super-wide-field TPLSM in vivo, it might be possible to image highly correlated activities in a RFA L5 neuronal soma and its axons projecting to CFA L1. In the mouse in which the AAV was injected only into the RFA, we moved a piezo objective positioner along the optical axis, and simultaneously rotated the device perpendicular to the optical axis to sequentially image the RFA L5 at a depth of 450 µm from the cortical surface and the CFA L1 at a depth of 25 µm at a frame rate of 4.4 Hz (Fig. 6a–d). The distribution of the CCs in the inferred spike events between pairs of RFA L5 neuronal somata and CFA L1 axonal boutons showed a Gaussian pattern with a center close to zero (Fig. 6e); however, a small separate cluster with CCs of around 0.75 was clearly detectable (Fig. 6e). The values of this cluster were equivalent to the CCs between pairs of axonal boutons derived from the putative same axon^25^ (gray histogram in Fig. 6e), and were much higher than the highest CCs (0.374) between pairs of neurons in the local RFA field. We found that one RFA neuron showed CCs of above 0.6 with multiple axonal boutons in the CFA, but that it had much lower CCs with all other axonal boutons; these high CCs corresponded to 6.5 s.d. of the mean of the CC distribution (Fig. 6f–h). This suggests that no neurons had high CCs with multiple boutons by chance. A pixel-wise correlation analysis showed highly correlated pixels forming shapes with axon-like line structures with boutons (Fig. 6i). Therefore, we consider that these pairings between this neuronal soma and the seven axonal boutons probably represent the same neuron. CCs in the inferred spike events of axonal boutons detected at the frame rate of 30 Hz were stably maintained, even when the imaging data were down-sampled to 5 Hz (*r* = 0.91 ± 0.02, *n* = 6 fields; Supplementary Fig. 7), which was similar to the actually used frame rate (4.4 Hz). Thus, when axonal labeling is sparse and the frequency at which uncorrelated spike events occur over short intervals is sparse, the dual-field imaging at different depths will be useful for functional mapping of long-range projections at a single-axon resolution.

### Dual-field imaging of mice performing a lever-pull task

**Fig. 7:**
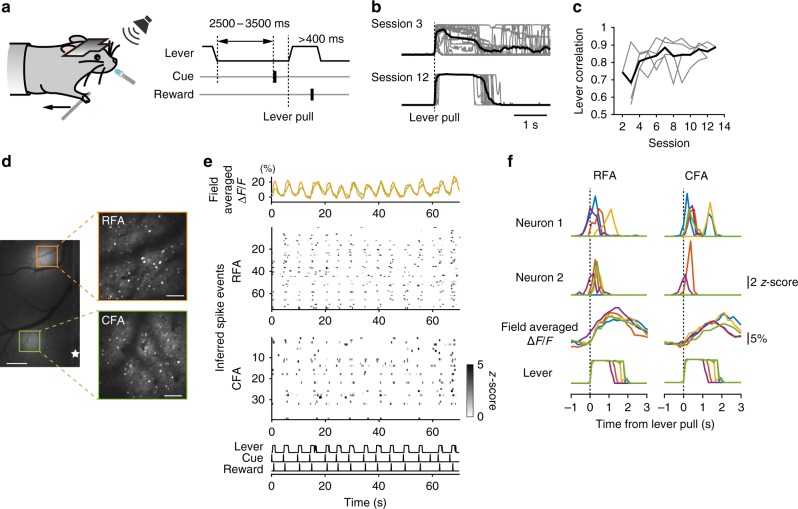
Dual-field imaging of the RFA and CFA during performance of a forelimb-movement task. **a** Schema of the sound-cued lever-pull task. To obtain a drop of water, the mouse needed to use its right forelimb to pull the lever 5 mm for a duration of 400 ms after presentation of a tone. **b** Example lever trajectories from sessions 3 and 12. Lever trajectories were aligned to the lever-pull onset. The first 20 trials of each session are shown. Gray lines indicate individual trials and black lines are the means. **c** Improvement in lever correlation during the task sessions. The sessions in which the mice performed a simpler task to associate the cue signal and licking (session 1 for one mouse and sessions 1 and 2 for three mice) were not plotted. **d** Left, an epifluorescence image of GCaMP6s-expressing neocortex. Orange (RFA) and green (CFA) squares indicate imaging fields of the dual-field two-photon imaging. The white star indicates the bregma. Scale bar, 500 µm. Right, averaged two-photon images of layer 2/3 neuronal somata in the RFA and CFA. Scale bar, 100 µm. **e** Representative neuronal activity of RFA and CFA somata during performance of the forelimb-movement task. Averaged fluorescence change of the whole imaging field (top; field-averaged Δ*F/F*), *z*-scored inferred spike events of neurons (middle), and lever trajectory, cue, and reward timings (bottom). **f** Traces of inferred spike events from four neurons numbered in **e**, field-averaged Δ*F/F*, and lever trajectory from five representative successful lever-pull trials. Each color (blue, red, yellow, green, and purple) indicates traces from the same trial

RFA and CFA are strongly related to forelimb movement and necessary for coordinated activity during performance of forelimb-movement tasks^19,28–32^. However, it is unknown whether trial-to-trial neuronal population activity in the RFA and CFA is mutually related or independent. To address this issue, we used super-wide-field TPLSM on mice performing a forelimb-movement task (Fig. 7a). In this task, the mice had to use their right forelimb to pull the lever within 1 s of a sound cue being presented, and then had to hold the lever for 400 ms to get a water reward. The sound cue was repeated at 2.5–3.5 s intervals after the lever was returned to its original position. As the training progressed, the reproducibility of the successful lever-pull trajectory increased and reached a plateau during training sessions 8–11 (Fig. 7b, c). Then, we conducted concurrent calcium imaging of L2/3 neurons that expressed GCaMP6s in the left RFA and left CFA during the task performance (Fig. 7d–f; 11 imaging sessions from four mice). We detected 25–105 neurons with calcium transients in each field (63 ± 7.0 neurons in RFA L2/3 and 42 ± 2.6 neurons in CFA L2/3, *n* = 11 imaging sessions), and more than half of them showed task-related inferred spike events (54.7 ± 6.7% in RFA L2/3 and 54.8 ± 3.4% in CFA L2/3, *n* = 11; see Methods for details). Although the field-averaged fluorescence change and lever trajectory were relatively stable across successful lever-pull trials (Fig. 7f; trial-to-trial correlation of field-averaged fluorescence change: 0.95 ± 0.01 in RFA L2/3 and 0.87 ± 0.02 in CFA L2/3; trial-to-trial correlation of lever trajectory, 0.89 ± 0.03, *n* = 11), each task-related neuron showed low trial-to-trial correlation for the inferred spike events (Fig. 7f; 0.27 ± 0.01 in RFA L2/3, *n* = 376; 0.22 ± 0.01 in CFA L2/3, *n* = 258). This suggests that the population activity in the high-dimensional space formed by multiple neurons was largely different across trials.

**Fig. 8:**
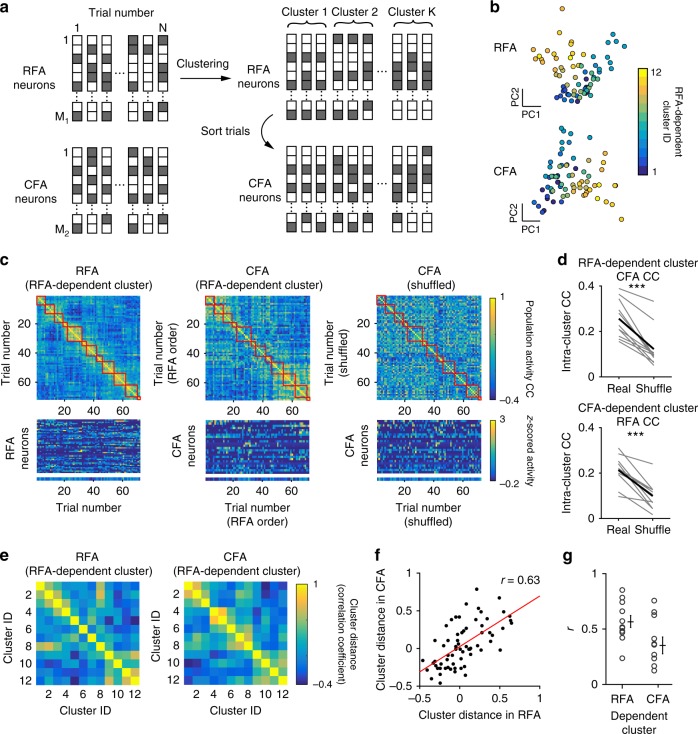
Relationships in trial-to-trial population activity between RFA and CFA. **a** Schematic of the analytical procedures. Successful trials were classified into multiple clusters based on the similarity of the RFA population activity. Then, the similarity of the CFA population activity within each cluster was also estimated. **b** Scatter plots of the first and second principal components of RFA (top) and CFA (bottom) population activity in each trial in an example imaging session. Face colors indicate the trial cluster numbers assigned from the RFA population activity. We show this plot only for display purpose. **c** Example correlation coefficient matrices in the population activity. In this imaging session, trials were classified into 12 clusters (red squares) based on the similarity of RFA population activity, and trials were sorted according to the cluster identity of the RFA (left and middle) and randomized order (right). The normalized time-averaged activity of each neuron and the field-averaged fluorescence changes in each trial are shown at the bottom. Left, matrix made from the RFA population activity. Middle, matrix made from the CFA population activity. Right, matrix made from the trial-shuffled CFA population activity. The imaging session was the same as in **b**. **d** Averaged trial-to-trial population activity CC within the clusters (intra-cluster CC). In each field, the CCs between pairs of trials within each cluster were averaged, and these values were averaged across clusters. Gray lines indicate individual pairs and black lines mean values. ***p < 0.001, paired *t* test (*n* = 11). **e** Example matrices of the distance between intra-cluster-averaged population activity. The imaging session was the same as in **b**. The distance was defined as the correlation coefficient in the averaged population activity between clusters. **f** Scatter plots of the distances between the same pairs of RFA and CFA clusters shown in **e**. The red line is a regression line and its correlation coefficient (*r*) is also shown. **g** The correlation coefficient (*r*) for the regression line between cluster distance in the RFA and CFA in each imaging session (*n* = 11). Crosses indicate mean ± s.e.m.

To examine whether the trial-to-trial population activity was similar between RFA and CFA, we calculated the time-averaged inferred spike events (from 0.46 s before to 1.85 s after the onset of each successful lever pull) of task-related neurons, and defined a vector with time-averaged activity of task-related neurons for each trial as the population activity. The population activity showed low trial-to-trial correlation (0.10 ± 0.018 in RFA L2/3 and 0.12 ± 0.026 in CFA L2/3, *n* = 11). The successful trials for each imaging session were classified into 8–14 clusters according to the similarity of their RFA population activity (RFA-dependent clusters; Fig. 8a–c; see Methods for details). If the population activity in the RFA and CFA covaried, the similarity of the CFA population activity within each cluster was also high, and the extent of the difference in the population activity between different clusters was similar between the RFA and CFA. As expected, the correlation for the CFA population activity within the clusters was higher than that between randomly chosen trials (Fig. 8c, d; *p* = 4.1 × 10^−5^; paired *t* test, *n* = 11). We obtained similar results when the cluster was determined according to the similarity of the CFA population activity among the successful trials (CFA-dependent cluster) and the RFA population activity within the clusters was examined (*p* = 6.1 × 10^−5^; paired *t* test, *n* = 11). Calculation of these correlations from the imaging data acquired at a frame rate of 5.4 Hz was rational because the CC was well preserved when the images acquired at 30 Hz were down-sampled to 5 Hz (*r* = 0.87 ± 0.02, *n* = 7 fields; Supplementary Fig. 8). The relative locations of trial clusters in the two-dimensional space of the RFA population activity appeared similar to those of the CFA population activity (Fig. 8b), and the distances between different clusters of RFA population activity correlated well with those between the different clusters of CFA population activity (Fig. 8e–g; see Methods for details). The cluster classification was not strongly related to similarities in lever-pull speed, lever trajectory, reaction time, or reward history within each cluster (Supplementary Fig. 9a, b; see Methods for details). Even within a small subset of clusters with high similarity for these properties, the correlations of these properties within each cluster were not different from those between different clusters (Supplementary Fig. 9c, d). In addition, the intra-cluster similarity in the population activity when the cluster was determined according to the similarity of the four behavioral properties (behavior-dependent cluster) was lower than those from RFA-dependent and CFA-dependent clusters (Supplementary Fig. 10). Trial clusters determined by population activity were not strongly related to patterns of similarity in the field-averaged fluorescence change within each cluster (Supplementary Fig. 9e–h). These results suggest that the reason RFA and CFA population activity covaried across trials was not simply because of trial-to-trial variabilities in behaviors or the net activity of the RFA and CFA, but was probably also because of the network dynamics embedded in the RFA and CFA.

## Discussion

In this work, we developed a novel micro-opto-mechanical device that is inserted into the post-objective space. This device added the ability to switch the FOV (e.g., in 50 ms for a 2.5 mm distance) of the microscope. Although this method is substantially different from other published methods for expanding the FOV, the features of two-photon imaging (deep imaging, subcellular imaging, and multiple-neuron imaging) were comparable to those of other methods using ultra-large-sized objectives^12,13^. Moreover, the maximum distance of >6 mm for in vivo imaging of different cortical areas exceeded the 3–5 mm distances described in previous studies.

A limitation of this method is that the axial resolution of approximately 9–12 µm was lower than normally achieved in two-photon microscopy (~2 µm). However, we believe this value to be sufficient to detect calcium transients from single neuronal somata because the soma size is approximately 10 µm and the infrequent occurrence of fluorescence changes (calcium transients) allows us to distinguish different activity patterns from partly overlapping neuronal somata^11,12^. In addition, when axons or dendrites were sparsely labeled, their calcium transients could be detected (Fig. 6 and Supplementary Fig. 5). If the WD of the objective could be lengthened, a larger microprism could be inserted to improve the spatial resolution. If the present spatial resolution is sufficient, the NA of the objective could be reduced to match the mirror area (from 0.6 to ~0.4). Low NA objectives generally have long WDs, which may allow us to increase the distance between the centers of the two FOVs, *d*_m–m_ × 2 × sin (*θ*/2).

Another limitation of this method is the dead time (40–80 ms) required to move the FOV (or rotate the mirror holder). The moving velocity could be increased by increasing the driving torque with a larger and higher-powered motor, although this may increase the vibration of the mirror holder. Alternatively, the dead time may be shortened by decreasing the inertia of the mirror holder. The upper aperture of the mirror holder could be made smaller if the gear that rotates it was set around the objective exit site; this would result in the mirror holder being lighter. In addition, as the distance between the centers of the two FOVs is *d*_m–m_ × 2 × sin (*θ*/2), an increase in *d*_m–m_ decreases *θ* and dead time, although the WD decreases.

Another limit of the method is that the imaging area is limited to the donut-shaped area and imaging of the whole donut-shaped and its center areas (circle with a diameter of ~6.4 mm) is impossible (Fig. 1h). However, by appropriately moving the center of the donut shape (i.e., the objective axis) and setting *θ*, any two FOVs can be sequentially imaged, although the edges of the FOVs may sometimes go un-imaged if the distance between the centers of these FOVs is less than the maximum of *d*_m–m_ × 2 × sin (*θ*/2) (5 mm when *d*_m–m_ is 2.5 mm). Sofroniew et al^13^. describe a 12 kHz resonant scanner that can image at 10 Mpixel s^−1^ (corresponding to four fields of 512 × 512 pixels at 9.5 Hz) and Stirman et al.^12^ describe temporal multiplexing devices that can image at 7.8 Mpixel s^−1^ (corresponding to two fields with 512 × 256 pixels at 30 Hz). With the pixel size of our system set to the same as these previous methods, we can image 2.6 Mpixel s^−1^ (corresponding to two fields of 512 × 512 pixels at 5 Hz). The systems of Sofroniew et al.^13^ and Stirman et al.^12^ can therefore, respectively, image ~3.8-fold and 3-fold larger numbers of neurons per second than our current method. We expect that our microscopy system with these additions could image at ~7.8 Mpixel s^−1^. If the super-wide-field microscopy is improved by incorporating the techniques described in these previous studies, the temporal resolution of the detected neuronal activity should be increased.

Using the super-wide-field microscopy, we demonstrated for the first time that the time-averaged population activity in the distal motor cortical areas co-varies across trials of a forelimb-movement task. Trial-to-trial correlations between neurons are thought to reflect patterns of synaptic inputs that are common to both and the reciprocal connections between them^33–35^. In fact, RFA and CFA possess reciprocal connections^27,36^ and receive common inputs from the thalamus^37^. The four behavioral properties and the net motor cortical activity were less related to the similarity of the population activity in the RFA (or CFA) within trial clusters than the population activity in the CFA (or RFA) was. These facts suggest that intrinsic brain properties such as neural fluctuation and the constrained subspace of the population activity^38–41^ may induce the simultaneous transition of population activity patterns in the RFA and CFA through cortico-cortical and subcortico-cortical pathways. However, the temporal pattern of the neuronal activity was not analyzed in this study, and it is reported that population activity is modulated by various components, such as movement reproducibility, reaction time, and reward history^42–44^. Recent studies suggest that body movements strongly modulate neuronal activity in rodents, even in the visual cortex^45,46^. Although it is difficult for two-photon calcium imaging at a frame rate of ≤30 Hz to detect spike synchronization and *γ* oscillation, such as those observed in the motor cortex^47–49^, improvement in the temporal resolution of the microscope as described above and the introduction of high-speed videography of orofacial behavior and postures would make super-wide-field microscopy a powerful tool for clarifying the relationships between time-evolving population activity in multiple areas and many body movements^44,50^.

The essential feature of our design lies in the use of the post-objective space. In other studies, optical elements have been inserted into the sample tissue. For example, insertion of a microprism into the rodent neocortex allowed other researchers to laterally image entire cortical layers^51^. A microprism was also inserted into deep fissures to observe the medial prefrontal cortex and medial entorhinal cortex^52,53^. In another study imaging mouse hippocampal neurons, the excitation light emitted from the objective was introduced to a GRIN lens that was embedded in the neocortical tissue above the hippocampus^54^. However, these studies used optical elements in a fixed manner. The use of a non-invasive movable optical element in the post-objective space brings several advantages that are not possessed by other methods. The first is modularity; our post-objective device does not require customization of the pre-objective space. Thus, it can be easily installed on a conventional TPLSM, and can also be removed to return the instrument to the original state. The second advantage is that it is aberration free. We used only mirrors as optical elements, which do not affect chromatic aberrations. Thus, the system preserved the original corrections for chromatic aberrations of the microscope and objective, although the FWHMs degraded ~20% from the center to the edge of the FOV. Indeed, we used 940 and 1100 nm to excite GCaMP6s and R-CaMP1.07, respectively. This means that our system is compatible with multicolor two-photon calcium imaging^6,7,55^. The third advantage is extensibility; introduction of other micro-optics may further expand the application of the post-objective space. For example, by replacing one micromirror with a micro-electro-mechanical systems mirror, it should be possible to flexibly tilt the image plane to remove optical aberration caused by the mismatch of the angle between the excitation light pathway and the glass window on each imaging cortical field. If a GRIN lens can be inserted between the mirror pair as a relay optical system, the mirror-to-mirror distance *d*_m–m_ could be extended further, regardless of the WD of the objective lens. These extensions may be useful when observing the broad (>1 cm) neocortex of non-human primates^56–58^. The post-objective space is a new frontier in microscope design.

## Methods

### Design and construction of the micro-opto-mechanical device

The micro-opto-mechanical device consisted of four parts: a pair of mirrors, a mirror holder, a rotation mount, and a pillar (Fig. 1c–g and Supplementary Fig. 1). A pair of right-angle prism mirrors with protected aluminum coatings (2.5 × 4 mm^2^; RPB3-2.5L04-550; Sigmakoki, Saitama, Japan) were embedded in the bottom of a cone-shaped holder (printed by a professional 3D printer; ProJetHD3500Plus; 3D Systems, SC, Rock Hill, USA; the STL file of the holder for 3D printing is added as Supplementary Data 1). The use of 3D printing technology enabled the fabrication of a lightweight (3.7 g) holder that fitted the objective shape and minimized interference with the head fixation plate attached to the mouse skull. The rotation mount was composed of a gear with an aperture of 32 mm diameter to rotate the upper circumference of the holder (module, 0.8; number of teeth, 80), a large diameter bearing (6809; NTN Corporation, Osaka, Japan), an intermediate gear (module, 0.8; number of teeth, 30), and a closed-loop stepper motor (AR46AA; Oriental Motor, Tokyo, Japan) with a gear (module, 0.8; number of teeth, 30). These parts were packed into a custom-machined aluminum case. The holder with the mirrors was attached to the rotation mount and installed under a water-immersion long-WD high-NA objective (XLPLN10XSVMP; ×10; WD, 8.0 mm; NA, 0.6; Olympus, Tokyo, Japan) connected to a commercially available two-photon microscope (FVMPE-RS; Olympus). The upper internal circumference of the holder surrounded, but did not contact, the circumference of the objective. The mount was supported by the pillar, independent of the microscope and the mouse head-fixing pole. The center position of the device was adjusted to fit with the optical axis of the objective using an *XYZ*-axis rack and pinion dovetail stage (TAR-70135; Sigmakoki) placed between the mount and the pillar. A small coverslip (No. 1 thickness; Matsunami Glass, Tokyo, Japan) was glued to the bottom of the holder to permit water filling under and above the bottom of the holder (protection glass). The moving speed and position of the rotation were regulated by a programmable motor driver (ARD-C; Oriental Motor) and synchronized by a program written with LabVIEW (National Instruments, Austin, TX, USA), which used transistor–transistor-logic signals of the image timing from the two-photon microscopy system.

### Estimation of the properties of super-wide-field TPLSM

A broadly tunable ultrafast laser (InSight DS Dual; Spectra Physics, San Francisco, CA, USA) at a wavelength of 940 nm was used for two-photon excitation. The beam shapes under the objective (Supplementary Fig. 2d) were measured by a beam analyzer (Super BeamAlyzer; Melles Griot, Carlsbad, CA, USA) with a detector.

To estimate the spatial resolution of the super-wide-field TPLSM (Fig. 2 and Supplementary Figs. 3, 4), fluorescent microbeads (0.5 µm; Fluoresbrite Yellow Green Microspheres; Polyscience, Warrington, PA, USA) were embedded in 0.5% low melting-point agarose (Agarose L; Nippon Gene, Tokyo, Japan) at a total concentration of 1.82 × 10^8^ beads ml^−1^. The sample was applied on a glass bottomed dish (No. 0 thickness; Matsunami Glass) and sealed with a coverslip to avoid drying. The beads were imaged through the bottom of the dish. The dish was mounted on a motorized *XYZ* stage (MP-285; Sutter Instruments, Novato, CA, USA) to precisely control the distance between the bottom surface of the glass of the dish and the bottom surface of the mirror holder (*d*_m–c_ in Fig. 2a and Supplementary Fig. 2). Therefore, *d*_m–c_ included the coverslip thickness of the dish (100 µm) and the thickness of the protection glass (150 µm). Fifty micrometer Z stacks of *XY* images containing a single fluorescent microbead were acquired with galvano scanners (spatial resolution: 0.249 × 0.249 × 0.5 µm^3^ pixel^−1^). Five frames were acquired at each focal plane and the average image was used. The images were imported into MATLAB, and FWHMs of individual beads were estimated. For the lateral point spread function, a plane with the maximum average intensity was used to obtain line profiles along the *X*-axis and *Y*-axis. For the axial point spread function, *XZ* and *YZ* images were created from the center of the bead, and line profiles along the directions in which beads were elongated were used^12^. Gaussian curves were fitted to the intensity profiles with the *fit* function of MATLAB to estimate the FWHMs. The axial FWHM was presented as an average of the FWHMs estimated from *XZ* and *YZ* images.

To estimate the speed and stability of the device rotation, fluorescent microbeads (2 µm; Fluoresbrite Yellow Green Microspheres; Polyscience) embedded in 4% agarose and sealed with a coverslip (No. 0 thickness; Matsunami Glass) were imaged.

### Animals

All animal experiments were approved by the Institutional Animal Care and Use Committee of the National Institutes of Natural Sciences and the Animal Experimental Committee of the University of Tokyo. Ten male C57BL/6 mice (aged 2 months) were used for the experiments. All mice were provided with food and water ad libitum and housed in a 12:12 h light–dark cycle, unless otherwise mentioned. Mice were anesthetized by intraperitoneal injection of ketamine (74 mg kg^−1^) and xylazine (10 mg kg^−1^) before an incision was made in the skin covering the neocortex. After the exposed skull was cleaned, a head plate^19^ (Tsukasa Giken, Sizuoka, Japan) was attached to the skull using dental cement (Fuji lute BC; GC, Tokyo, Japan; and Bistite II; Tokuyama Dental, Tokyo, Japan). The surface of the intact skull was coated with dental adhesive resin cement (Super bond; Sun Medical, Siga, Japan) to prevent drying. The number of mice per cage was 2–5 before the head plate was attached, but the mice were single-caged after attachment to avoid damage to the head plate and the glass window.

### Virus production

For imaging of R-CaMP1.07, GCaMP3 DNA of pAAV-human synapsin I promoter (hSyn)-GCaMP3-WPRE-hGH polyA^5^ was replaced with R-CaMP1.07 DNA from pN1-R-CaMP1.07 vector construct^17^. rAAV2/1-hSyn-R-CaMP1.07 (1.3 × 10^13^ vector genomes ml^−1^) was produced with pAAV2-1 and purified as described previously^59^. rAAV2/1-SynI-GCaMP6s (1.3 × 10^13^ vector genomes ml^−1^) was obtained from the University of Pennsylvania Gene Therapy Program Vector Core.

### Virus injection

One hour before surgery, dexamethasone sodium phosphate (1.32 mg kg^−1^), d-mannitol (2.0 g kg^−1^; to reduce brain swelling and promote homogeneous virus expression^60^), the antibiotics sulfadiazine (24 mg kg^−1^) and trimethoprim (4.8 mg kg^−1^), and the anti-inflammatory carprofen (6 mg kg^−1^) were administered intraperitoneally. Isoflurane (1%) inhalation was used for anesthesia throughout surgery. An 8 × 6 mm^2^ (for left and right somatosensory cortex imaging; centered at the bregma) or a 2 × 4 mm^2^ (for motor cortex imaging; centered at 1.0 mm anterior to the bregma and 1.2 mm left of the midline) rectangular craniotomy was made. Before virus injection, a pulled glass pipette (broken and beveled to an outer diameter of 30 μm) and a 5 μl Hamilton syringe were back-filled with mineral oil and front-loaded with virus solution. For dual-field imaging (Fig. 4), three sites in the left and right somatosensory cortex (centered at 0.5 mm posterior and 2.5 mm lateral to the bregma and 0.3 mm ventral from the cortical surface) were each injected with 200 nl rAAV2/1-SynI-R-CaMP1.07. For stitch imaging (Fig. 5), ten sites were each injected with 200 nl rAAV2/1-SynI-R-CaMP1.07 according to the craniotomy. For imaging of somata or the dendrites in the RFA and CFA (Fig. 7 and Supplementary Fig. 5), one site in the RFA (2.5 mm anterior and 0.8 mm lateral to the bregma and 0.5 mm ventral from the cortical surface) and one site in the CFA (0.5 mm anterior and 1.2 mm lateral to the bregma and 0.5 mm ventral from the cortical surface) were each injected with 200 nl rAAV2/1-SynI-GCaMP6s. For imaging of neuronal somata and axons (Fig. 6 and Supplementary Fig. 7) and imaging of deep neuronal somata (Supplementary Fig. 6), one site in the RFA (2.5 mm anterior and 0.8 mm lateral to the bregma and 0.5 mm ventral from the cortical surface) was injected with 100 nl rAAV2/1-SynI-GCaMP6s. The pipette was then withdrawn, a single-layer (8 × 6 mm^2^ No. 2 thickness; Matsunami glass) or double-layer glass window was pressed onto the brain surface, and the edge was sealed with cyanoacrylate glue (Vetbond; 3M, St. Paul, MN, USA) and dental adhesive resin cement (Super bond). The double-layer glass window was constructed by joining a 3 × 5 mm^2^ rectangular glass coverslip (No. 0 thickness; Matsunami Glass) and a 2 × 4 mm^2^ rectangular glass coverslip (No. 4 thickness; Matsunami Glass) with a UV-curing optical adhesive (NOR-61; Norland Products, Cranbury, NJ, USA). Mice were then returned to their home cages. Before the imaging experiments were performed, the mice were allowed to recover and express sufficient quantities of R-CaMP1.07 or GCaMP6s for 2–4 weeks.

### In vivo imaging with super-wide-field TPLSM

Mice were head-restrained under the mirror holder and the objective. Before imaging, a two-axis goniometer attached to the head-fixing device was adjusted so that the glass window on the brain and the objective lens were nearly horizontal. The wavelength of the laser was set at 940 nm for GCaMP6s imaging and 1100 nm for R-CaMP1.07 imaging. Fluorescence emission was directly collected with a GaAsP photomultiplier tube (Hamamatsu Photonics, Sizuoka, Japan) after separation from the excitation beam path through a 690 nm shortpass dichroic mirror (Olympus). The laser intensity under the device was 30–62 mW for imaging of L1 dendrites, L1 axons, L2/3 somata, and L5 somata. A piezo objective positioner (P-725KHDS; Physik Instrumente, Karlsruhe, Germany) was controlled using microscope control software (FV30S-SW; Olympus) to rapidly change the imaging field along the optical axis.

The procedure for imaging two target regions was as follows. First, the relative coordinates of the two regions in the horizontal direction were measured using an epifluorescence image with a low magnification objective (LMPlanFl, ×5; Olympus) or by moving an *XY* stage from the center of one region to that of the other. By solving a simple trigonometric equation, the rotation angles for imaging of the two target regions were determined. It is necessary to avoid putting the dental cement on the portion of the skull below which the center of the donut shape may be set. After setting the angle of the rotation mount to one target region, the FOV was moved to the center of the target region. Parallelism between the mirror holder and glass window was important. For example, when a large glass window of 8 mm (used in Fig. 4) is set at a 5° tilt relative to the horizontal plane, *d*_m–c_ at the edge of the glass at the bottom side is 0.7 mm longer than at the edge of the glass at the top side. This extension to *d*_m–c_ decreases *d*_o–m_, so that the beam entering and passing through the mirrors, and therefore also the spatial resolution, is decreased. Thus, the mirror holder and glass window should be kept parallel as much as possible to maintain them in close proximity. After the bottom of the mirror holder and the glass window were placed as close as possible, images were acquired while the timings of rotation, imaging, and piezo movements were controlled by a program written with LabVIEW.

### Image processing

Analyses were performed using MATLAB (2015b; Mathworks, Natick, MA, USA) and ImageJ (National Institute of Health, Bethesda, MD, USA). Lateral displacements of acquired image sequences were corrected using TurboReg^61^ (for imaging of the neuronal soma) and a non-rigid motion correction algorithm^62^ (for imaging of dendrites and axons). After the motion correction, the CNMF algorithm^18^ was used to determine regions of interest (ROIs). The algorithm extracts a time course of inferred calcium transients (temporal component) with their spatial position and shape (spatial component) by minimizing a residual between the raw image sequence and an image sequence generated by multiplying the spatial and temporal components. The algorithm also performs deconvolution of calcium transients at the same time, and we refer to these as “inferred spike events.” When the activity was extracted from the neuronal somata, the components were selected according to their size (>50 µm^2^) and the eccentricity of the ellipse fitted to the ROI (>0.5). This procedure was effective in removing extracted apical dendrites in L2/3 planes. In Fig. 4g and Supplementary Fig. 7b, fluorescence signals calculated by multiplying the spatial component and the raw image sequence were used to show the quality of the raw imaging. In both cases, for the calculation of the trace of the relative fluorescence changes (Δ*F/F*), the baseline (*F*) was estimated with the eighth percentile value of the whole acquired time series of the fluorescence signals. In the analysis of the stitch imaging, 1500 continuous frames acquired over a duration of 8.1 min were used; these were obtained from an awake mouse without task training. An image stitching script written for ImageJ (“Pairwise stitching”^63^) was used to stitch the three adjacent fields together. Neural activity in overlapping regions was extracted from image sequences in only one of the fields to avoid double counting. Inferred spike events were filtered with a Gaussian kernel (100 ms standard deviation) using a zero-phase digital filter (MATLAB “filtfilt” function) and were used to calculate pairwise correlations.

### Analysis of the dual-field soma–axon imaging

In the analysis of the dual-field soma–axon imaging, 3000 continuous frames acquired over 11.4 min were used; these were obtained from an awake mouse without task training. A movable task device lever (described below) was placed near the right forelimb. The mouse usually grasped a lever while maintaining a relaxed posture (Fig. 1g). To reduce potential pseudo-correlations via body movements, non-lever moving periods (excluding periods from 1 s before to 1 s after the lever was moved) were concatenated and analyzed (2749 frames, 10.4 min). For the pairwise correlation analysis, inferred spike events were filtered with a Gaussian kernel (500 ms standard deviation). To extract robust pairs, only ROIs that showed >10 peak events during the period were used. The peak events were identified from the filtered inferred spike events using the *findpeaks* function of MATLAB with a threshold of 2 s.d. For the analysis of the effect of the frame rate on pairwise CCs (Supplementary Fig. 7), original 30 Hz images were down-sampled by skipping frames by factors of 2, 6, 10, 15, 30, 60, and 120 frames. To estimate the effect of down-sampling from the same ROIs, spatial components (i.e., positions of the ROIs) identified by the CNMF algorithm applied to the 30 Hz images were used to extract temporal components (neural activity) from the down-sampled images.

### Sound-cued lever-pull task

The task was modified from a previously described self-initiated lever-pull task^5,19^. Briefly, mice with head plates were inserted into a body chamber and their heads were fixed in the task device. The mice had been water deprived in their home cages and were maintained at 80–85% of their normal weights throughout the experiments. To obtain a 4 μl drop of water delivered from a spout near their mouths, the mice were trained to use their right forelimbs to pull the lever 5 mm for 400 ms within 1 s of the onset of a 100 ms 11 kHz pure tone. The next sound cue was given 2.5–3.5 s after the lever was returned to its original position. In the first session (one mouse) or the first and second sessions (three mice), the reward was delivered when the mice licked the spout within 1 s of the end of the tone after the cue was presented, irrespective of any lever movement. Next, the forelimb lever-pull task was started, with the duration of lever pull being extended from 1 to 200 ms, and then finally to 400 ms during the training sessions. This learning curriculum ensured that the success rate was kept high from the beginning (82 ± 14% on session 2; 85 ± 6.7% on the final training day before imaging, *n* = 4). The mice were trained for 8–11 days (1 h per day) to finally pull the lever for 400 ms with high lever correlation (Fig. 7c). This lever correlation was defined as the mean CC of the individual lever trajectories from –0.5 to 2.0 s of lever-pull initiation against the averaged trajectory of all successful trials. A program written with LabVIEW was used to control the task device. After training, the mice performed the task under the microscope. The sound and vibration noises generated by the holder rotation appeared to have a negligible effect on the task performance, as indicated by the absence of a significant difference in the reaction time to the sound cue (*p* = 0.29; paired *t* test; 178 ± 26 ms during imaging; 208 ± 48 ms on the final training day before imaging, *n* = 4), the lever correlation (*p* = 0.55; paired *t* test; 0.88 ± 0.02 during imaging; 0.84 ± 0.06 on the final training day before imaging, *n* = 4), and the success rate (*p* = 0.43; paired *t* test; 89 ± 5.9% during imaging; 85 ± 6.7% on the final training day before imaging, *n* = 4).

### Analysis of the dual-field imaging of the RFA and CFA

Continuously recorded 9.3 min periods of dual-field imaging data from mice performing the sound-cued lever-pull task were used to analyze the dual-field imaging of the RFA and CFA. The frame rate for this imaging was 5.4 Hz. The mice successfully completed 80 ± 5.7 trials (*n* = 11) during the imaging session. Successful trials that did not show a short lever-pull movement before the initiation of the successful lever pull (400 ms pull) after the cue was presented, and with reaction times of 100–500 ms, were used for the analysis (81.0% of all successful trials; 65 ± 5.6 trials, *n* = 11). The duration of each successful lever-pull movement, which was defined as the time from pull initiation to lever return, was 1.5 ± 0.11 s (*n* = 11). The inferred spike events and behavioral data were resampled with the interp1 function of MATLAB according to the entire dual-field frame acquisition timings. The resulting sampling rate (resampled rate) was 10.8 Hz. Neurons were classified as movement related when their inferred spike events during the whole movement-related period (−5 to 20 resampled frames from the successful lever-pull onset) were significantly larger than those of the whole resting period (−25 to −10 resampled frames from the successful lever-pull onset; *p* < 0.05; Wilcoxon's rank-sum test). The proportions of movement-related neurons were comparable to those obtained in our past data acquired during a self-initiated lever-pull task (51.4% in RFA L2/3 and 45.6% in CFA L2/3)^19^. To calculate the trial-to-trial correlation of the population activity, traces of *z*-scored inferred spike events of movement-related neurons during the movement-related period were realigned to a matrix with dimensions of cells × time × trials. We used *z*-scored activity of each neuron to avoid the effect of possible outliers, such as neurons with exceptionally high activity rate^41,43,64,65^. The time dimension of this array was then averaged to generate a trial-to-trial population activity matrix and used to calculate a trial-to-trial population activity correlation coefficient (population activity CC). The field-averaged fluorescence was generated as the mean fluorescence intensity of each frame across the entire FOV. For the calculation of the trace of field-averaged relative fluorescence changes (Δ*F/F*), the baseline was estimated with the eighth percentile value of the whole acquired time series. The trial-to-trial correlation of field-averaged Δ*F/F*, lever trajectory, and the inferred spike events were calculated as the average of the CCs between the trial-averaged trace and individual trial traces during the movement-related periods.

Although we took care to balance the injections, we found a small but significant difference in the decay time constants of the calcium transients between RFA and CFA neurons (*p* = 0.0058; Wilcoxon's rank-sum test; 1.40 ± 0.05 s in RFA and 1.22 ± 0.03 s in CFA, *n* = 11 imaging sessions). This difference could possibly cause differences in the inference of the neuronal activity. However, the mean, standard deviation, and maximum values of the inferred spike events during the non-lever moving periods in each field were not significantly different between RFA and CFA neurons (mean: 0.0439 ± 0.0062 in RFA and 0.046 ± 0.0062 in CFA; standard deviation: 0.2627 ± 0.0323 in RFA and 0.2656 ± 0.0256 in CFA; maximum values: 4.5737 ± 0.4760 in RFA and 4.6497 ± 0.8653 in CFA; all *p* > 0.55; Wilcoxon's rank-sum test; *n* = 11). Too strong expression of GCaMP causes aberrant responses in the visual cortex and might change the trial-to-trial variability^66^. However, within the range of our experiment, the mean CC in the population activity between pairs of trials did not correlate with the decay time constants (*p* *=* 0.60; *r* = 0.12; Pearson’s CC; *n* = 22 RFA and CFA fields), or with the number of neurons detected in each field (*p* *=* 0.78; *r* = 0.06; Pearson’s CC; *n* = 22 RFA and CFA fields). Thus, we concluded that the differences in the fluorescence expression between the RFA and CFA neurons did not have large effects on the results regarding the correlation analyses.

### Clustering analysis of trial-to-trial population activity

Affinity propagation was used for the clustering of trials (Fig. 8a, c) because it does not have an initial value dependency and there is no requirement to define the cluster number in advance, in contrast to other clustering algorithms such as *K*-means^44,67^. This algorithm has two inputs, a distance matrix and a similarity measure, which define a general range of the number of clusters. We calculated the distance as the CCs between all pairs of trial population activity vectors. When Euclidean distance was used, the intra-cluster CC in the CFA population from the RFA-dependent clusters was significantly higher than that from shuffled trials (0.23 ± 0.02; shuffled trials, 0.12 ± 0.03; *p* = 2.9 × 10^–4^, *n* = 11, paired *t* test). For the similarity measure (a parameter of the algorithm), the median of the elements of the distance matrix was used according to ref ^67^. The affinity propagation classified the trials into 8–14 clusters per imaging session (10.4 ± 0.6, *n* = 11) when RFA population activity was used (RFA-dependent clusters). When the 1st, 10th, 30th, or 70th percentile were used as the parameter instead of the median value, the numbers of clusters were 7.5 ± 0.7, 7.8 ± 0.6, 10.3 ± 0.6, or 12.3 ± 0.7, respectively (*n* = 11), with the major results being similar to those obtained using the median value. To visualize high-dimensional population activity (Fig. 8b), principal component analysis was applied to the trial-to-trial population activity matrix of one field, and the top two components were plotted. In the example shown in Fig. 8b, only 31 and 33% of the variance of the respective RFA and CFA population activity is explained by these components; therefore, the distance between a pair of clusters does not reflect the actual distance between a pair of the vectors of the population activity (46 RFA and 24 CFA neurons; *n* = 71 trials). To calculate intra-cluster CCs (Fig. 8d), the CCs for the CFA population activity between pairs of trials within each RFA-dependent cluster were averaged. A shuffled intra-cluster CC was calculated by shuffling the trial labels, and the average value of 1000 shuffles was used. To calculate the inter-cluster distance matrix (Fig. 8e), RFA (or CFA) population activity was trial averaged within each cluster, and the CCs between the averaged activity of different clusters were calculated. The CC is only sensitive to the angle between the population vectors and is insensitive to the scaling^68^. We used this measure to focus our analysis on changes in patterns of active neuron groups, rather than the total strength of the population activity. However, when we used Euclidean distance as a measure of population activity distance^69^, we obtained similar CCs in the inter-cluster distance between RFA and CFA population activity (*r* = 0.60 ± 0.05 for RFA-dependent cluster and *r* = 0.50 ± 0.07 for CFA-dependent cluster, *n* = 11) to those shown in Fig. 8g. In addition, when non-deconvolved Δ*F/F* traces were used to represent the population activity instead of inferred spike rates, the intra-cluster CC in the CFA population from the RFA-dependent cluster (0.26 ± 0.02, *n* = 11) was significantly higher than that from shuffled data (0.06 ± 0.01; *p* = 5.3 × 10^–6^, paired *t* test), the intra-cluster CC in the RFA population from the CFA-dependent cluster (0.27 ± 0.03, *n* = 11) was significantly higher than that from shuffled data (0.10 ± 0.02; *p* = 8.5 × 10^–5^, paired *t* test), and the correlation coefficient for the regression line between cluster distance in the RFA and CFA was 0.57 ± 0.05 (*n* = 11) for the RFA-dependent cluster and 0.48 ± 0.08 for the CFA-dependent cluster. Thus, we concluded that the results shown in Fig. 8 were not due to an artifact caused by the selection of the analytical parameters.

To examine the behavioral effects on the population activity clusters, we calculated four behavioral properties (Supplementary Fig. 9). Lever-pull speed at lever-pull initiation (lever-pull speed) was defined as the maximum slope of the lever trajectory in ± 100 ms of lever-pull initiation. Lever correlation was defined as the CC between the individual lever trajectory from –0.43 to 1.85 s of lever-pull initiation and the averaged trajectory of all successful trials during the same imaging session. Reaction time was defined as the time from the cue initiation to lever-pull initiation. Time from previous reward was defined as the time from the last reward delivery to lever-pull initiation. To cluster the trials according to four behavioral properties (Supplementary Fig. 10), affinity propagation was used as described above.

To analyze the effect of the frame rate on the CC of the population activity between trials (Supplementary Fig. 8), the original 30 Hz images were down-sampled by skipping frames by factors of 2, 6, 10, 15, 30, 60, and 120 frames. To compare the effect of down-sampling from the same ROIs, the spatial components (i.e., positions of the ROIs) identified by the CNMF algorithm applied to the 30 Hz images were used to extract temporal components (neuronal activity) from the down-sampled images. Population activity CCs were calculated from these down-sampled activities using the method described above.

### Statistics

Statistical analyses were performed using MATLAB. Data are presented as mean ± s.e.m., unless otherwise stated. Wilcoxon's rank-sum tests, paired *t* tests, and random permutation tests were used for statistical comparisons. We did not employ statistical methods to predetermine sample sizes; however, sample sizes were estimated on the basis of methodically comparable previous laboratory experiments^5^ and are similar to those generally employed in the field. No randomization was used in the analysis.

### Code availability

The MATLAB codes for the CNMF algorithm^18^ and the non-rigid motion correction algorithm^62^ are available on the code author’s GitHub repository (https://github.com/epnev/). The ImageJ plugin for the Turboreg algorithm^61^ is available on its author’s website (http://bigwww.epfl.ch/thevenaz/turboreg/). The MATLAB code for the Affinity propagation algorithm^67^ is available on its author’s website (http://www.psi.toronto.edu/index.php?q=affinity%20propagation). Other codes used in the analyses are available from the corresponding authors upon reasonable request.

## Electronic supplementary material

Supplementary Information

Peer Review File

Description of Additional Supplementary Files

Supplementary Data 1

Supplementary Movie 1

Supplementary Movie 2

Supplementary Movie 3

## Data Availability

The data that support the findings of this study are available from the corresponding authors upon reasonable request.
