# Supplementary material for: Super-wide-field two-photon imaging with a micro-optical device moving in post-objective space
Source: Nat Commun. 2018 Sep 3;9:3550. doi: 10.1038/s41467-018-06058-8 (PMC6120955; doi:10.1038/s41467-018-06058-8)
Supplement: Supplementary file 1 — Supplementary Information [file 41467_2018_6058_MOESM1_ESM.pdf]

# Supplementary information

Super-wide-field two-photon imaging with a micro-optical device moving in post-objective space

Terada et al.

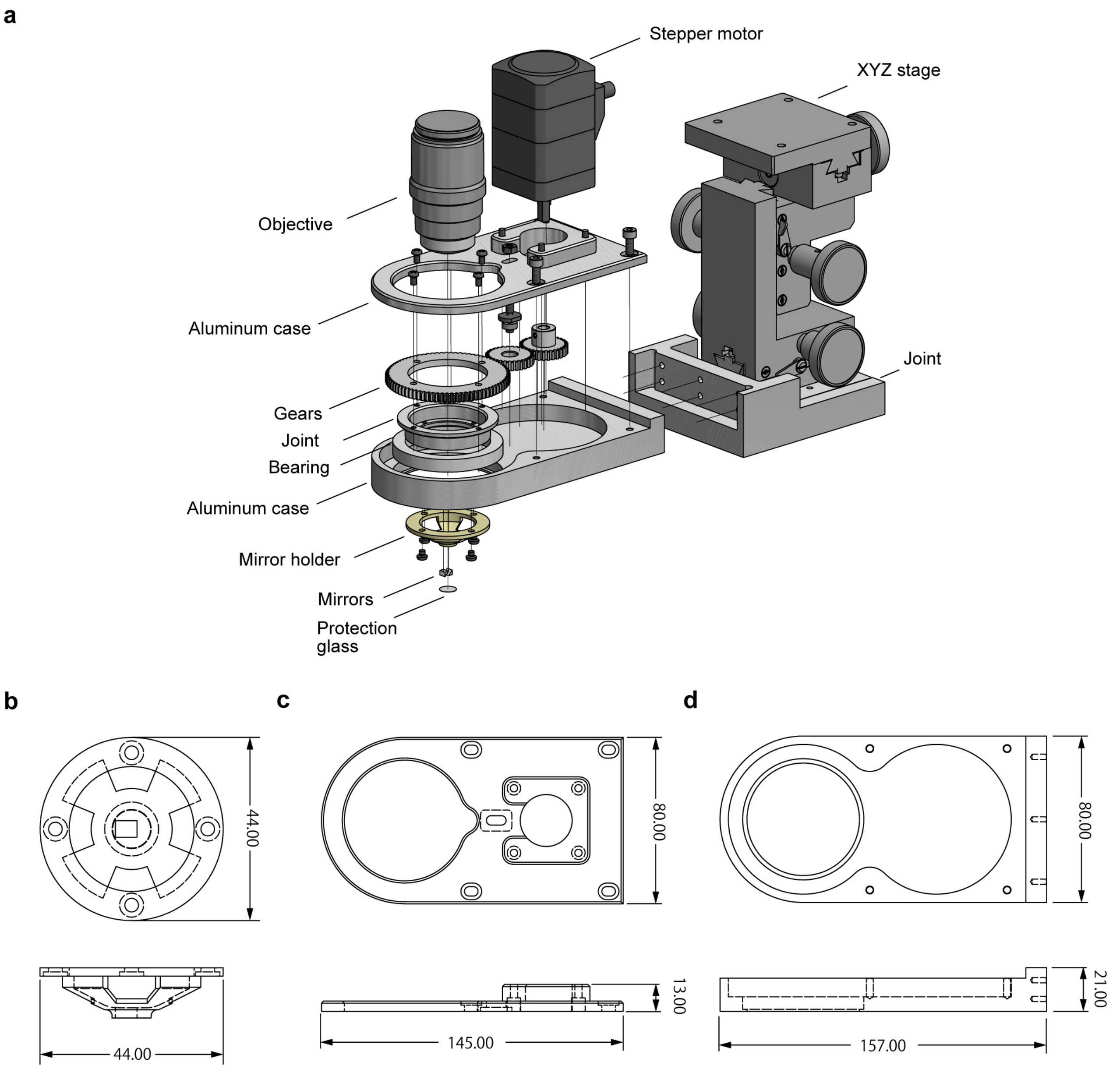


**Supplementary Figure 1** | Drawings of the micro-opto-mechanical device.

**a** Exploded view of the device. See Methods for gear ratios and model number information. **b–d** Top and side views of the mirror holder (**b**) and upper (**c**) and lower (**d**) parts of the aluminum case. Units are millimeters.

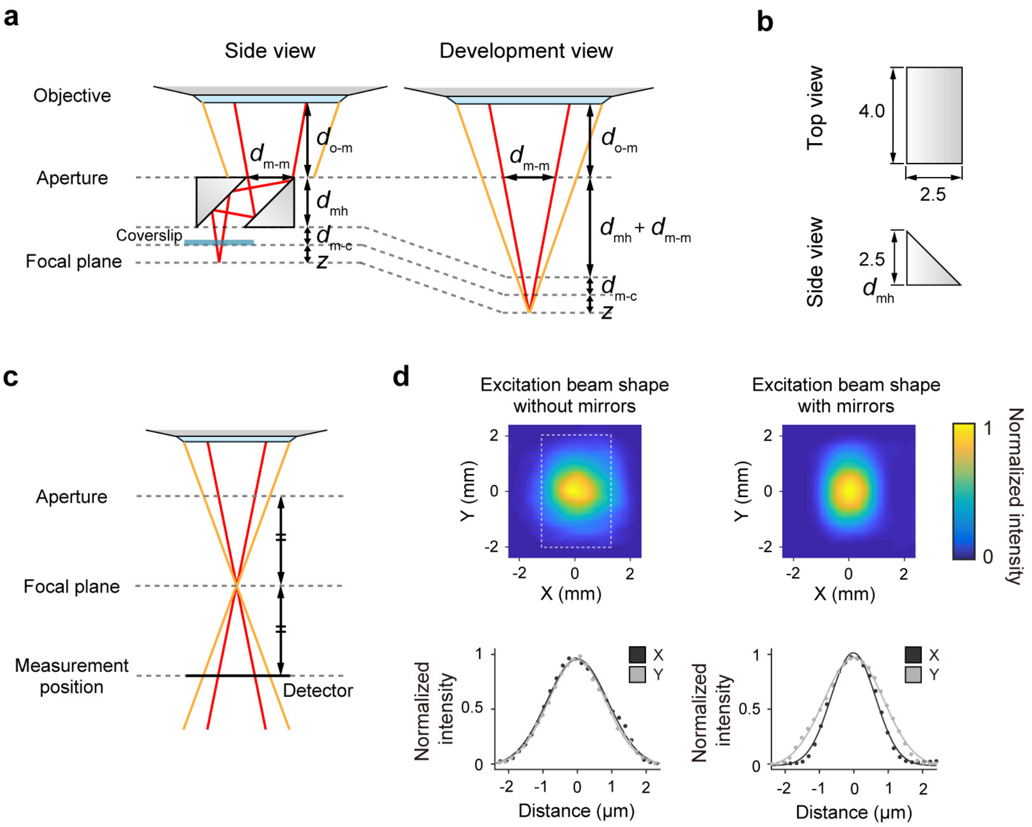


**Supplementary Figure 2** | Laser beam pathway and shape after the micro-opto-mechanical device.

**a** Schematic illustration of the excitation beam through a mirror pair. $d_{m\text{–}m}$ is the distance between the mirror pair. $d_{o\text{–}m}$ is the distance between the objective exit and upper surface of the mirrors. $d_{m\text{–}c}$ is the distance between the bottom surface of the mirror holder and the bottom surface of the coverslip. $z$ is the distance between the bottom surface of the coverslip and the focal plane. The mirror size was smaller than the laser beam entering the mirror (orange), so that the outer area (between orange and red lines) of the laser beam was cut. **b** Top and side views of the mirror. $d_{mh}$ is the height of the mirror. The units are millimeters. **c** Schematic illustration of the beam shape measurement. The beam shapes were measured at a conjugate plane of the aperture under conditions with and without the mirrors. When $d_{mh} = 2.5$ mm, $d_{m\text{–}m} = 2.5$ mm, $d_{m\text{–}c} = 1.15$ mm, and $z = 0$ mm, the distance from the focus to the opening of the mirror is 6.15 mm. The coverslip was not inserted in this measurement. As a water-immersion objective working in air was used for the measurement, the beam shape was measured at 4.6 mm (6.15 mm divided by the refractive index difference 1.33) under the focus (measurement position). **d** Excitation beam shapes without mirrors (i.e., at the device aperture; left) and with mirrors (i.e., after the aperture; right). Dotted rectangle indicates the device aperture created by the pair of 2.5 × 4.0 mm mirrors. Intensity line profiles along the X and Y axes were plotted with Gaussian curves. Almost all beams were transmitted in the Y axis direction, but clipping occurred in the X axis direction, resulting in an elliptical beam (FWHMs; 2.09 mm for X axis and 1.96 mm for Y-axis at the aperture; 1.52 mm for X axis and 2.06 mm for Y-axis after the aperture). The measured transmittance of the laser power after passing through the mirrors was 66%.

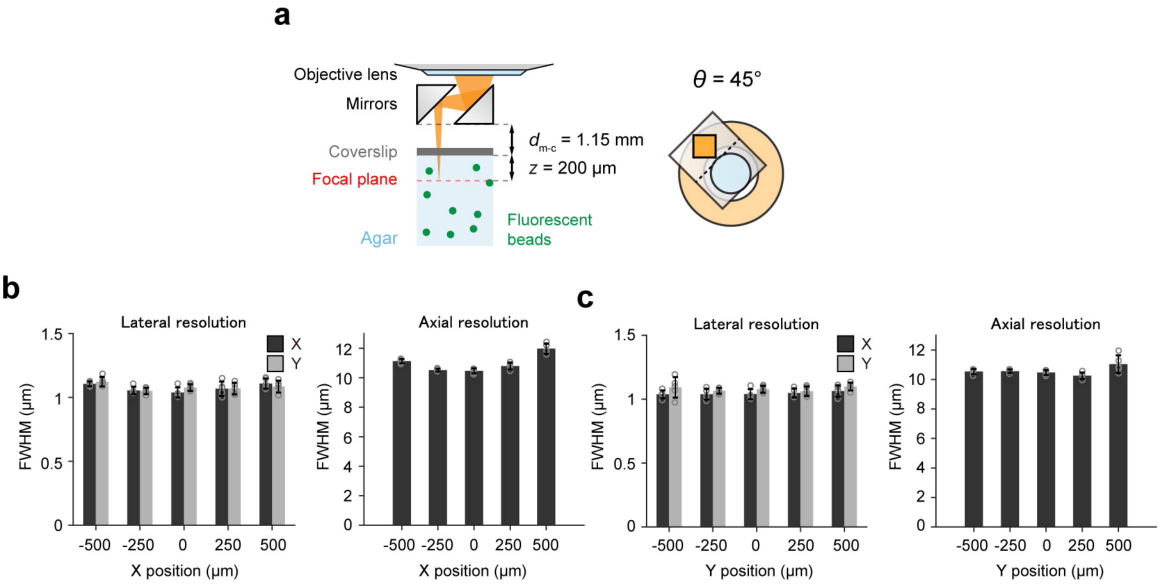


**Supplementary Figure 3** | FWHMs of the beads at nine different positions in the FOV at $\theta$ of 45°. **a** Measurement configuration to estimate the spatial resolution. $d_{m\text{–}c}$ was set at 1.15 mm, $z$ was 200 μm, and $\theta$ was 45°. **b, c** Lateral and axial FWHMs of the beads along the X (**b**) and Y (**c**) axes. Five different beads were measured for each point. Gray dots indicate individual bead measurements. Data are plotted as mean ± s.d.

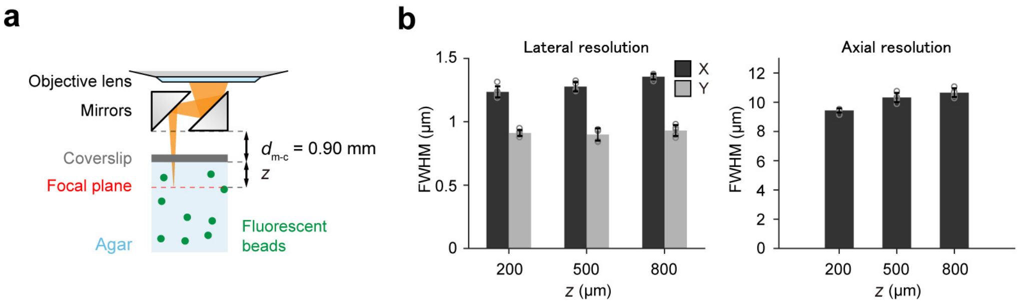


**Supplementary Figure 4** | FWHMs of the beads at three different depths with $d_{m\text{–}c}$ of 0.90 mm.

**a** Measurement configuration to estimate the spatial resolution. $d_{m\text{–}c}$ was set at 0.90 mm. **b** Lateral and axial FWHMs at the center of the FOV at three different values of $z$. Five different beads were measured for each point. Gray dots indicate individual bead measurements. Data are plotted as mean ± s.d.

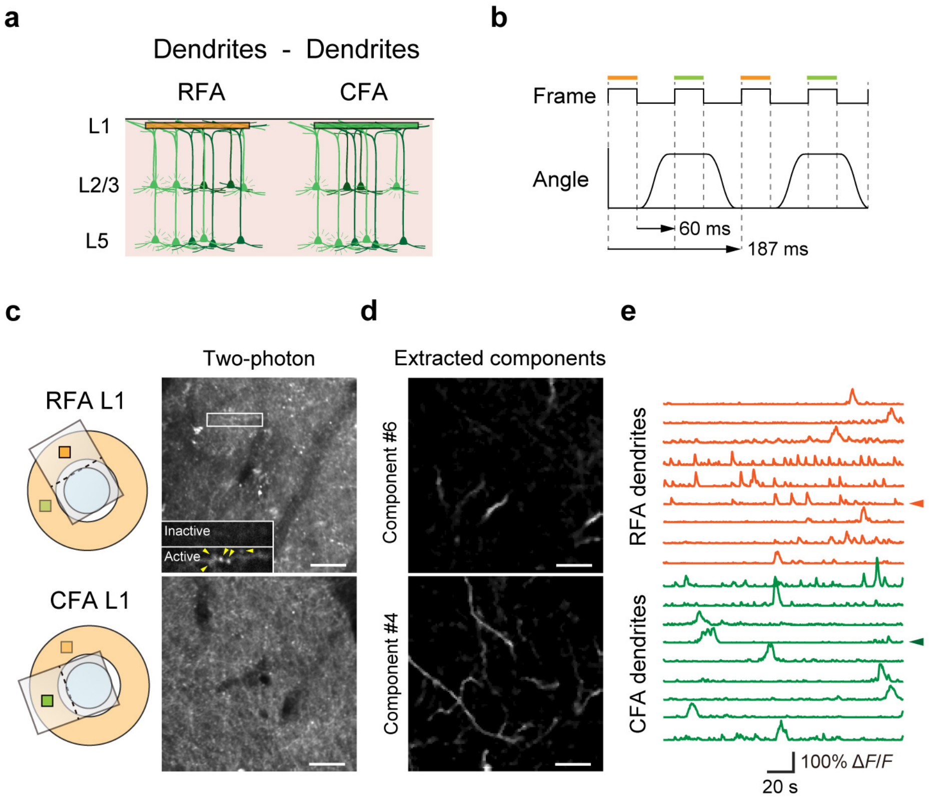


**Supplementary Figure 5** | Dual-field imaging of L1 dendritic activity in the RFA and CFA.
**a** Schematic illustration of imaged areas (green, CFA; orange, RFA). **b** Timing chart showing the scanning frame and rotation angle during four consecutive imaging frames. **c** Left, schematic illustration of the FOV and device position at each imaging frame. Right, averaged two-photon images of GCaMP6s-expressing dendrites and/or axons. The depth of the fields in RFA L1 and CFA L1 was 25 µm. The inset shows a dendritic branch with dendritic spines (arrowheads) at inactivated and activated frames (average of ten consecutive frames; cropped from the white rectangle). Scale bar, 20 µm. **d** Representative spatial components extracted with the constrained non-negative matrix factorization algorithm. Each component properly captured the structure of the dendritic branches. Scale bar, 20 µm. **e** Representative Δ*F*/*F* traces of nine components from each field. Arrowheads indicate Δ*F*/*F* traces corresponding to the spatial components shown in **d**.

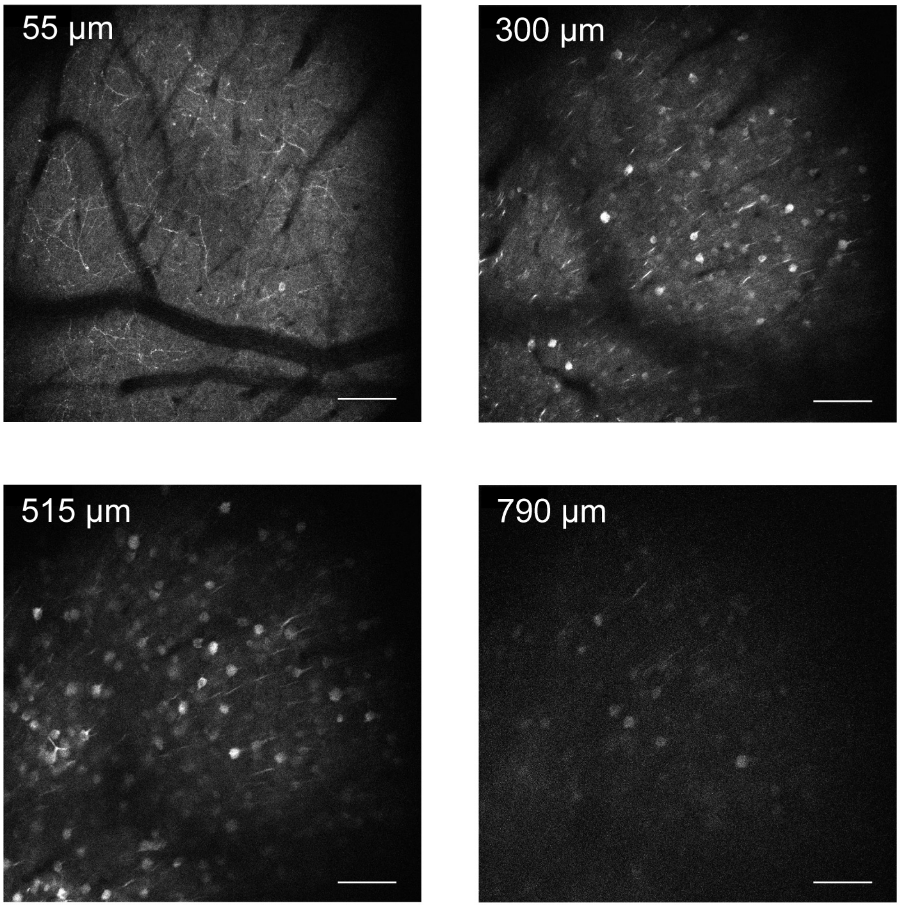


**Supplementary Figure 6** | *In vivo* two-photon imaging of GCaMP6s-expressing neurons through the device with no rotation.

Four representative fields at different depths taken from XYZ images of the RFA of an awake mouse. GCaMP6s was injected into the RFA. The depth from the cortical surface is shown in the top left of each image. Scale bar, 100 µm.

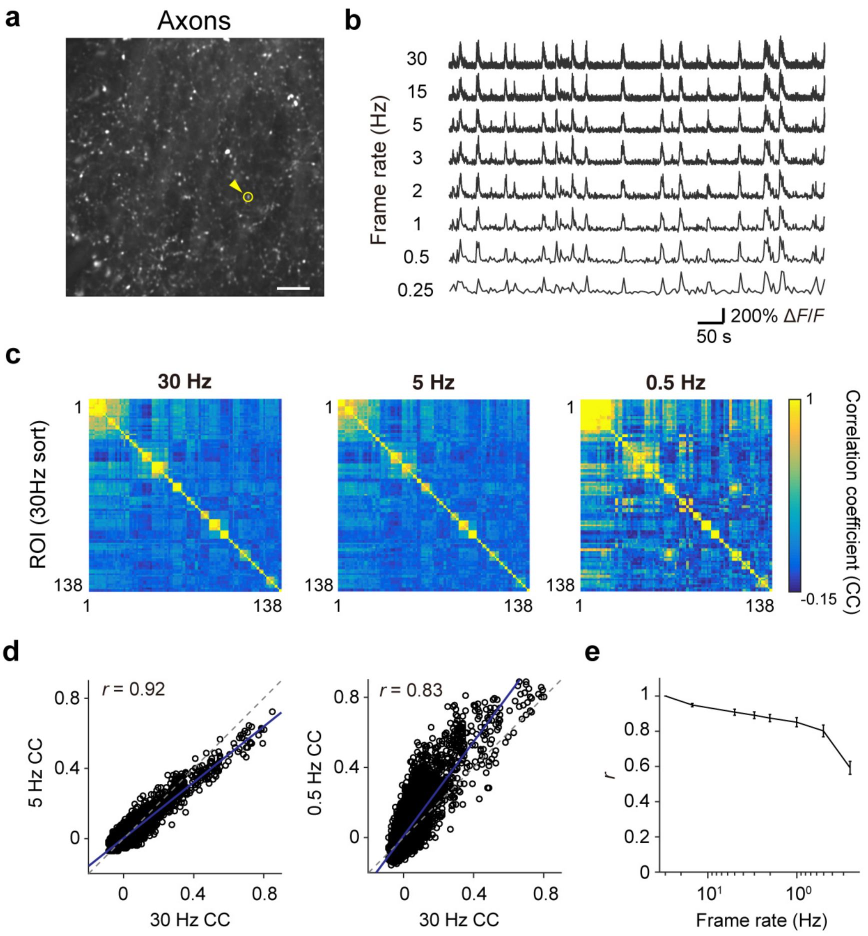


**Supplementary Figure 7** | Effect of the frame rate on correlation analysis of axonal boutons.
**a** An averaged two-photon image of GCaMP6s-expressing axons projecting from the RFA. The image was acquired in a CFA L1 field through the device with no rotation. Frame rate was 30 Hz. Scale bar, 20 µm. **b** Traces of raw fluorescence signals of the ROI area shown in **a** at eight frame rates. Only the trace at 30 Hz was acquired, and after imaging it was down-sampled to the traces shown in gray by skipping frames by factors of 2, 6, 10, 15, 30, 60, and 120 frames. **c–e** Effect of down-sampling on the correlation analysis. **c** Matrices of pairwise correlation coefficients (CC) between detected axonal boutons in one field at each of the three frame rates (30, 5, and 0.5 Hz). The matrix was arranged according to the orders of the hierarchal clusters estimated from the CCs at 30 Hz. **d** Scatter plots of CCs in the same pairs of ROIs at 30 Hz and 5 Hz (left), or at 30 Hz and 0.5 Hz (right; 9453 pairs of ROIs in a field). Blue lines are regression lines. The correlation coefficients (*r*) are also shown. **e** The correlation coefficient (*r*) for the CCs between 30 Hz and down-sampled data (15, 5, 3, 2, 1, 0.5, and 0.25 Hz; *n* = 6 fields from two mice).

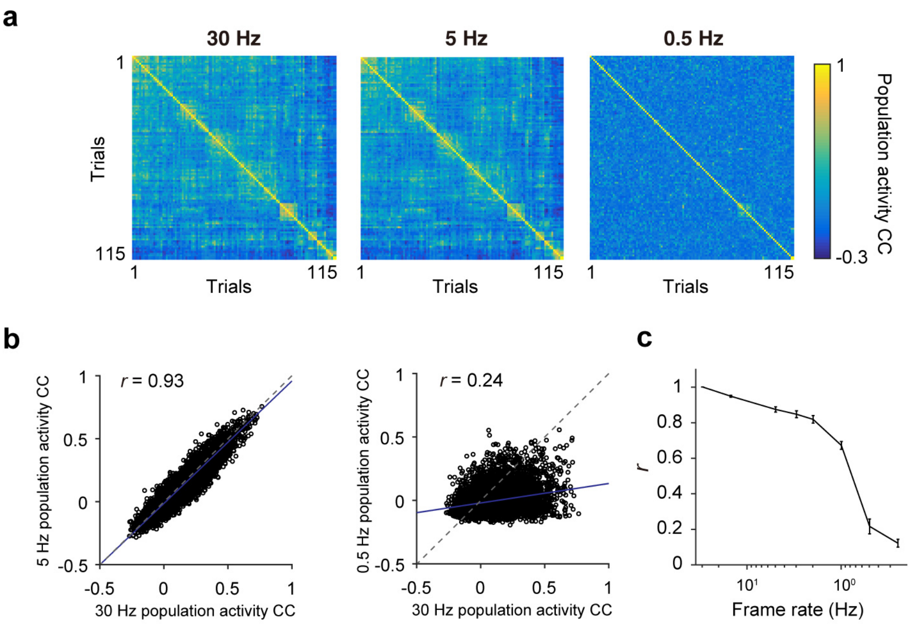


**Supplementary Figure 8** | Effect of frame rate on the trial-to-trial correlation in the population activity. Single field neuronal activity during performance of the forelimb-movement task was recorded at 30 Hz from RFA L2/3 or CFA L2/3. After imaging, it was down-sampled by skipping frames by factors of 2, 6, 10, 15, 30, 60, and 120 frames. **a** Representative matrices of CCs in the population activity between trials (population CCs) in one RFA field at each of three frame rates (30, 5, and 0.5 Hz). The matrix was arranged according to the orders of the hierarchal clusters estimated from the population activity CCs at 30 Hz. **b** Scatter plots of population activity CCs in the same pairs of ROIs at 30 Hz and 5 Hz (left), or at 30 Hz and 0.5 Hz (right; 6555 pairs of trials in a field). Blue lines are regression lines. The correlation coefficients (*r*) are also shown. **c** The correlation coefficient (*r*) for the population activity CCs between 30 Hz and down-sampled data (15, 5, 3, 2, 1, 0.5, and 0.25 Hz; three RFA fields and four CFA fields from four mice).

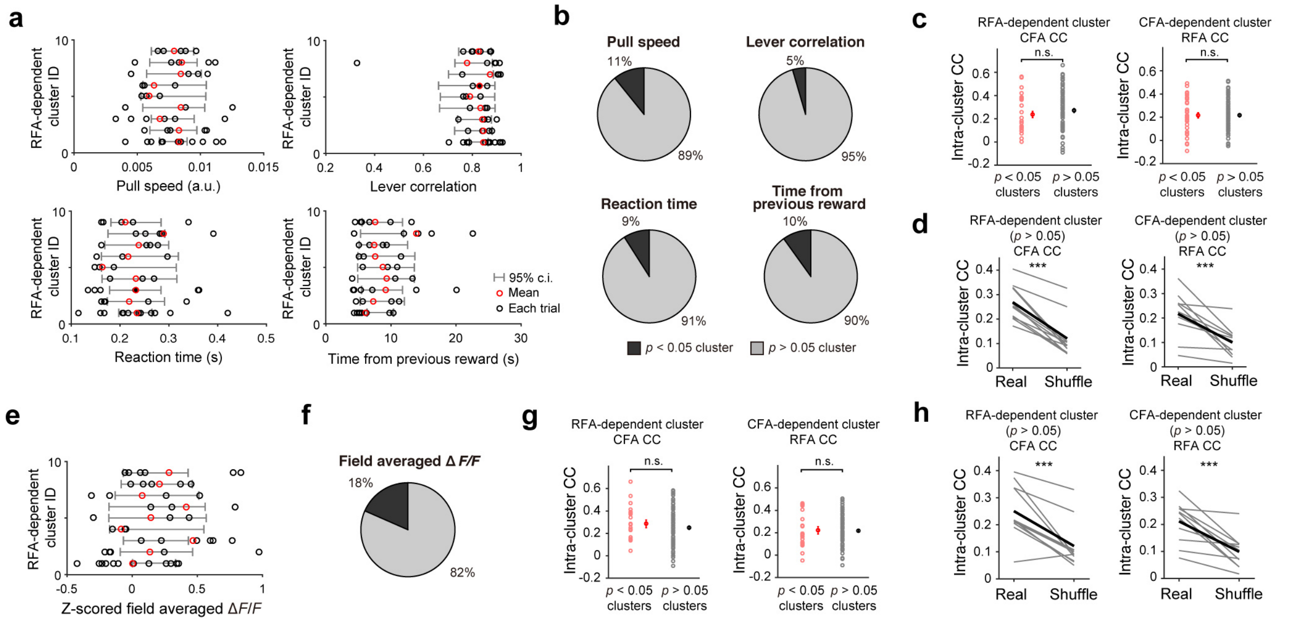


**Supplementary Figure 9** | Trial-to-trial correlation in the population activity did not strongly reflect the trial-to-trial correlation of behavioral variables or net activity.

**a** Distributions of four behavioral variables in nine RFA-dependent clusters in an example imaging session. The four behavioral variables were lever-pull speed, similarity of lever trajectory, reaction time, and time from previous reward (see Methods for details). Black dots indicate the values of the corresponding behavioral variable for each trial, red dots indicate the means, and gray bars indicate the 95% confidence intervals. To estimate the 95% confidence intervals, null distributions were generated by calculating the average value of trial-shuffled behavioral variables in each cluster for 2000 times. **b** Proportions of behavior-modulated clusters. The total number of RFA-dependent clusters was 114 from 11 imaging sessions. For each behavioral variable, the cluster with the mean of the behavioral variable outside the confidence intervals ($p < 0.05$ cluster) was classified as a behavior-modulated cluster. **c** Intra-cluster CCs for the population activity within behavior-modulated clusters (red; significant for at least one behavioral variable; $n = 36$ clusters) and within the other clusters (gray; non-significant for any behavioral variable; $n = 78$ clusters). Left, intra-cluster CCs in the CFA population activity from RFA-dependent clusters. Right, intra-cluster CCs in the RFA population activity from CFA-dependent clusters. Each dot indicates the mean intra-cluster CC for each cluster. If the trial-to-trial variability in the population activity strongly represents the trial-to-trial variability in behavioral variables, the intra-cluster CCs within behavior-modulated clusters should be higher than those within the other clusters. However, they did not differ between behavior-modulated clusters and the other clusters. **d** Field-averaged intra-cluster CCs in non-behavior-modulated clusters (68% and 67% of clusters in RFA- and CFA-dependent clusters, respectively). Gray lines indicate individual pairs and black lines indicate means. ***: $p < 0.001$, paired $t$-test ($n = 11$). This result indicates that the similarity between trial pairs of the population activity, even within each non-behavior-modulated cluster, was higher than that between randomly assigned pairs of the population activity. **e–h** The same analysis as in **a–d** with field-averaged fluorescence change.

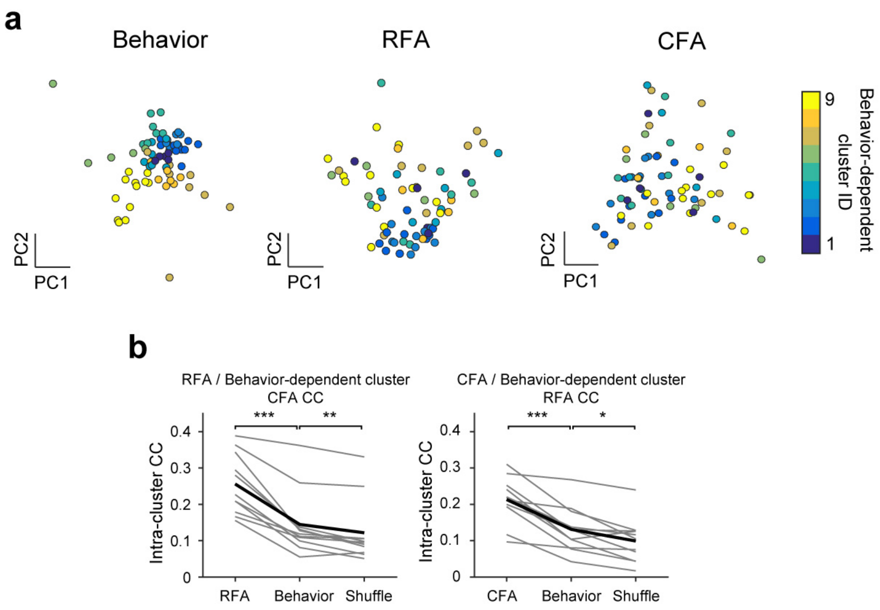


**Supplementary Figure 10** | Intra-cluster CCs were lower in behavior-dependent clustering than population activity-dependent clustering.

The behavior-dependent cluster was determined by the affinity propagation with the median of the elements of the distance matrix. **a** Scatter plots of the first and second principle components of the four behavioral variables (left), RFA population activity (middle), and CFA population activity (right) in each trial in the same imaging session as shown in Fig. 8b. Face colors indicate the trial cluster numbers assigned from the behavioral variables. RFA and CFA population activities were not well separated by the behavior-dependent clustering. **b** Left, field-averaged intra-cluster CCs in the CFA population activity from RFA-dependent cluster, behavior-dependent cluster, and shuffled data based on the behavior-dependent cluster. Right, field-averaged intra-cluster CCs in the RFA population activity from CFA-dependent cluster, behavior-dependent cluster, and shuffled data based on the behavior-dependent cluster. ***: $p < 0.001$, **: $p < 0.01$,*: $p < 0.05$, multiple paired $t$-test with Bonferroni correction ($n$ = 11).
