# Supplementary material for: Super-wide-field two-photon imaging with a micro-optical device moving in post-objective space
Source: Nat Commun. 2018 Sep 3;9:3550. doi: 10.1038/s41467-018-06058-8 (PMC6120955; doi:10.1038/s41467-018-06058-8)
Supplement: Supplementary file 3 — Description of Additional Supplementary Files [file 41467_2018_6058_MOESM3_ESM.pdf]

Description of Additional Supplementary Files:

Supplementary Data 1 | The STL file of the mirror holder for 3D printing.

Supplementary Movie 1 | Two-photon imaging of fluorescent microbeads during switching of the FOVs.
The images were continuously acquired at 236 frames/s by cropping the FOV to 57 × 512 pixels. The size of the image is 18 × 158 μm.

Supplementary Movie 2 | Dual-field imaging of left and right forelimb-related primary somatosensory cortices.
Motion-corrected movie of R-CaMP1.07-expressing neurons in left and right forelimb-related primary somatosensory cortical areas. The movie plays at 26× the true speed. The FOV size is 909 × 909 μm.

Supplementary Movie 3 | Stitch imaging of the motor cortex.
Motion-corrected movie of R-CaMP1.07-expressing neurons in the mouse motor cortex at 32× the true speed. The size of the stitched FOV is 1.2 × 3.5 mm. Overlapping regions were averaged.
