# Supplementary material for: Super-wide-field two-photon imaging with a micro-optical device moving in post-objective space
Source: Nat Commun. 2018 Sep 3;9:3550. doi: 10.1038/s41467-018-06058-8 (PMC6120955; doi:10.1038/s41467-018-06058-8)
Supplement: Supplementary file 2 — Peer Review File [file 41467_2018_6058_MOESM2_ESM.pdf]

Reviewers' comments:

Reviewer #1 (Remarks to the Author):

This paper from Terada et al. described an interesting and innovative optical technique to expand the size of the field of view of a 2-photon microscope by an order of magnitude, which appears to be useful for in vivo brain imaging. They attached a rotary micro-mirror device between the objective and the specimen. A pair of micro-mirrors bent the optical axis away from the optical axis and thus allows the authors to reach a region that cannot be usually reached. By rotating the micro-mirrors rapidly (~10 millisecond), they succeeded in pseudo-simultaneously imaging multiple locations in the cortex separated by more than millimeters with the temporal resolution of sub-seconds. The resolution of the image is reported to be ~1 um in lateral and ~10 um in axial directions, which are sufficiently high for performing Ca^{2+} imaging in dendrites, axons and somata.

While there are several reports achieving super-wide field of view using custom optics, the described technique would cost much less and can be applied to many commercial systems.

I would think that the system would have a certain niche for researchers who want to image neurons in multiple areas in the brain.

Specific comments

1. One major drawback of the described technique is a large "dead time" between image acquisitions due to the slow rotary movement of the mirrors. Thus, the temporal fraction in which photons are collected is smaller. This limits the number of neurons one can simultaneously image. The authors should discuss this drawback (and potential solutions), and compare the number of neurons one can simultaneously image with other imaging solutions.

2. p13, The authors discuss another reported wide-area system and claim that "however, the imaging of activity at depths of > 200 μm from the cortical surface and sub-cellular imaging are very difficult." Is this a speculation? Please provide optical/physical explanations of why their system can go deeper (or simply delete the statement). I think that the depth penetration would depend a lot more on sample prep rather than difference in optical settings. As the authors describe, it is much easier to image deeper in the cortex for sparsely labeled samples ---- but this is not the advantage of their new optical system.

Reviewer #2 (Remarks to the Author):

This is an innovative device that enables new neuroscience experiments. Its relative ease of implementation should interest a broad cross section of neuroscientists. The manuscript has been carefully prepared, clearly written, and the figures and materials completely describe the device. In its current state, the manuscript is nearly ready for publication.

Conventional 2-photon microscopes offer high resolution over very small fields of view. This is a problem because one often needs to simultaneously measure neural activity in two cells that are further apart than the field-of-view is wide. It takes too long to move the microscope or the preparation, which are both significant masses (considerable inertia). Several technological advances have been introduced in recent years to address this problem. All of them are complex and expensive, typically requiring entirely new systems. In this work, the authors present an elegant solution: move the field of view using mirrors AFTER the objective. This relatively low inertia device enables sub-

second sampling of areas in an annulus around the center. The annulus has a diameter much larger than the objective's natural field-of-view.

Although this solution is conceptually simple, there was a considerable amount of engineering that went into it to make the positioning reproducible, as fast as possible, and preserve as much fidelity of the imaging as possible (brightness, numerical aperture). Furthermore, the authors discuss some interesting extensions that are possible with this technology/approach.

Major points:
(1)
There are limitations to this device, and it would be good to expand the discussion of these a bit.

a.
The switching time is relatively slow, limiting frame rates to ~ 5 frames/sec. However, there are plenty of experiments that can be performed adequately using that acquisition speed, as the authors demonstrate. This is already adequately discussed.

b.
The NA is also quite low, but it is still usable. This could be discussed a bit more. Maybe one more sentence.

Also: It is unclear exactly what the NA is. It looks like ~0.28 according to Fig 1e. However, in that caption for that figure, the authors say it is 0.42. In the results section (line 107) it is stated estimated to be 0.36. To users, the precise value isn't so important because, empirically, it works. However, the confusion does a disservice to the paper. Please clear this up.

c.
The access area is limited to an annulus. This limitation isn't adequately discussed. The neurons of interest have to occur somewhere on the annulus, and this can be a significant limitation in some applications.

To be clear, I am strongly supportive of publication. I am only asking for a slight elaboration on points b and c, and a clarification on point b.

(2)
The authors share most, but not all, of the information necessary to facilitate replication of their device. One omission is the mechanical design. The 3D printing files should be shared, as well as information for the custom machined aluminum case mentioned in the methods. It would also be helpful to see a photograph of the device installed on a microscope as it would be for an experiment.

Minor points:

It's nice to cite colleagues, but it isn't necessary to compare this work to one photon approaches (ref. 30). If one was to do so, then there would be a lot to add, including SCAPE, lightfield imaging, etc. It simplifies the paper if only multiphoton approaches that work > 200 um deep in the neocortex are compared.

Reviewer #3 (Remarks to the Author):

This manuscript describes an interesting idea and its implementation: using a pair of mirrors

positioned after a long-working-distance objective, to rapidly steer the field of view of a 2p microscope to different locations beyond the normal field of view (FOV) of the objective. However, the improvement in FOV, at least as demonstrated, appears to be moderate (6.2 mm vs 5 mm reported), and this improvement is at the expense of greatly reduced NA and poorer axial resolution (12 µm vs 4.1 µm reported) and is associated with a large dead area at the center. Most of biological experiment demonstrations except Figure 3 can be accommodated by existing microscope designs. There also seems to other limitations of the current design (working distance/geometry). Some parameters remained to be reported/tested/discussed.

Specific comments:
1) What is the working distance (after the apparatus)? In figure 1d/e, it indicates that working distance is 1 mm. This is very short. Considering this: for a large glass window 7 mm in size (that is what this microscope is intended for): a 5 degree tilt of the window relative to optical axis would mean 0.6 mm, or most of the working distance. Also, in order to imaging a little bit deeper into tissue, the pair of mirror elements has to nearly directly touch the glass window, which causes many problems, and will be difficult for an average biologist. In the reviewer's experience, even going from a 3 mm to 2 mm working distance (in some available objectives) greatly reduced the usability. Although the element may be moved more upwards closer to the objective to give longer working distance, this maneuver appears to further reduce the NA, which is already very low even at the current design.
2) Along the line of the comments above, please provide a few real pictures showing the size, position of the apparatus relative to the microscope and the mouse. This will help the reader to understand the implementation and practicality issues.
3) What is the resolution/NA at the edge of FOV? While for all objectives, the performance at edge is poorer than center, the reviewer worries that the need to pass beam through the small aperture may further reduced NA at the edge with the current design, and the resolution/light collection may be poor. These factors may reduce the practical FOV.
4) The area at the center that cannot be imaged is quite big, occupying ~40-60% of the area depending on the practical FOV at a given position. How does this affect experimental designs? What experimental limitations come with this property? Some discussions on this topic are needed.
5) The use of an aperture that is not round/square, as shown in figure 1g, means that the NAs for x and y are different. How does this affect the resolution and/or the point spread function? Some discussions would be nice.
6) Figures 1f and 1g are confusing. The authors show an "inferred" beam size. Can this be replaced by a directly measurement? Is there an explanation that the beam shape is square? Also, by using a rectangular aperture as large as the beam area, when the aperture is rotated to a different angle, would part of the aperture not covered by the beam (and this would decrease NA)?

Reviewer #4 (Remarks to the Author):

Comments about the biology in the submission "Super-wide-field two-photon imaging with a micro-optical device 2 moving in post-objective space".

The authors utilize their new wide field imaging approach in attempts to learn something about inter-area connections and correlations. They attempt to use their microscope and correlation in calcium activity to trace single axons in vivo. While I think this is an interesting approach, the authors fail to convince me that they actually accomplished this goal. As a matter of fact, the only conclusion they make is "probably represented the same neuron". No alternative evidence is given that they did trace an axon. Since this method is based on correlations in activity, the slow imaging speed is also a significant problem, leading to potential false positives.

The other use case the authors attempted was looking at dynamics between cortical areas with cellular and subcellular resolution. Again, this is important but I do not think we learned much new from this approach. Interarea comparisons have already been made between cortical areas without cellular resolution. Here, the cellular resolution is used to look at correlations between neurons, but again the very slow imaging speeds limits the conclusions about observed correlations. Controls for other differences between areas also need to be performed, such as differences in somata depth, differences in expression level, differences in calcium buffering, and differences in action potential sensitivity. Comparisons between RFA and CFA have been done by the labs of Whishaw, Murphy, and He, all of which are not cited in this manuscript. This work needs to be put in context with the literature.

We thank the reviewers for their careful consideration of our manuscript and their constructive comments. Our detailed responses to the reviewers' comments are provided below:

Reviewers' comments:

Reviewer #1 (Remarks to the Author):

This paper from Terada et al. described an interesting and innovative optical technique to expand the size of the field of view of a 2-photon microscope by an order of magnitude, which appears to be useful for in vivo brain imaging. They attached a rotary micro-mirror device between the objective and the specimen. A pair of micro-mirrors bent the optical axis away from the optical axis and thus allows the authors to reach a region that cannot be usually reached. By rotating the micro-mirrors rapidly (~10 millisecond), they succeeded in pseudo-simultaneously imaging multiple locations in the cortex separated by more than millimeters with the temporal resolution of sub-seconds. The resolution of the image is reported to be ~1 um in lateral and ~10 um in axial directions, which are sufficiently high for performing Ca2+ imaging in dendrites, axons and somata.

While there are several reports achieving super-wide field of view using custom optics, the described technique would cost much less and can be applied to many commercial systems.

I would think that the system would have a certain niche for researchers who want to image neurons in multiple areas in the brain.

Specific comments

1. One major drawback of the described technique is a large "dead time" between image acquisitions due to the slow rotary movement of the mirrors. Thus, the temporal fraction in which photons are collected is smaller. This limits the number of neurons one can simultaneously image. The authors should discuss this drawback (and potential solutions), and compare the number of neurons one can simultaneously image with other imaging solutions.

We have added a discussion on the limit of the dead time and potential solutions to overcome this limit, and have compared the area imaged per second (because this determines the number of neurons that can be simultaneously detected) with other studies, as follows (lines 315–340):

"A limitation of this method is the dead time (40–80 ms) required to move the FOV (or rotate the mirror holder). The moving velocity could be increased by increasing the driving torque with a larger and higher-powered motor, although this may increase the vibration of the mirror holder. Alternatively, the dead time may be shortened by decreasing the inertia of the mirror holder. The upper aperture of the mirror holder could be made smaller if the gear that rotates it was set around the objective exit site; this would result in the mirror holder being lighter. In addition, as the distance between the centers of the two FOVs is $d_{m-m} \times 2 \times \sin(\theta/2)$, an increase in d_{m-m} decreases θ and dead time, although the working distance decreases. Another limit of the method is that the imaging area is limited to the donut-shaped area and imaging of the whole donut-shaped and its center areas (circle with a diameter of ~6.4 mm) is impossible (Fig. 1h). However, by appropriately moving the center of the donut-shape (i.e., the objective axis) and setting θ, any two FOVs can be sequentially imaged although the edges of the FOVs may sometimes go un-imaged if the distance between the centers of these FOVs is less than the maximum of $d_{m-m} \times 2 \times \sin(\theta/2)$ (5 mm when d_{m-m} is 2.5 mm). Sofrnoiew et al.[13] describe a 12 kHz resonant scanner that can image at 10 Mpixel/s (corresponding to four fields of 512 × 512 pixels at 9.5 Hz) and Stirman[12] et al. describe temporal multiplexing devices that can image at 7.8 Mpixel/s (corresponding to two fields with 512 × 256 pixels at 30 Hz). With the pixel size of our system set to the same as these previous methods, we can image 2.6 Mpixel/s (corresponding to two fields of 512 × 512 pixels at 5 Hz). The systems of Sofrnoiew et al. and Stirman et al. can therefore respectively image ~3.8-fold and 3-fold larger numbers of neurons per second than our current method. We expect that our microscopy system with these additions could image at ~7.8 Mpixel/s. If the super-wide-field microscopy is improved by incorporating the techniques described in these previous studies, the temporal resolution of the detected neuronal activity should be increased."

2. p13, The authors discuss another reported wide-area system and claim that “however, the imaging of activity at depths of > 200 μm from the cortical surface and sub-cellular imaging are very difficult.” Is this a speculation? Please provide optical/physical explanations of why their system can go deeper (or simply delete the statement). I think that the depth penetration would depend a lot more on sample prep rather than difference in optical settings. As the authors describe, it is much easier to image deeper in the cortex for sparsely labeled samples ---- but this is not the advantage of their new optical system.

We used the term “> 200 μm” according to the following sentence: “As one-photon Ca^{2+} imaging is limited to ~150–200 mm depths into brain tissue (Hamel et al., 2015)” in Kim et al. (Cell Reports 17, 3385–3394, 2016). We agree with the reviewer’s comments that depth penetration largely depends on the sample preparation. We have removed the comparison between two-photon imaging and one-photon imaging from the current manuscript.

Reviewer #2 (Remarks to the Author):

This is an innovative device that enables new neuroscience experiments. Its relative ease of implementation should interest a broad cross section of neuroscientists. The manuscript has been carefully prepared, clearly written, and the figures and materials completely describe the device. In its current state, the manuscript is nearly ready for publication.

Conventional 2-photon microscopes offer high resolution over very small fields of view. This is a problem because one often needs to simultaneously measure neural activity in two cells that are further apart than the field-of-view is wide. It takes too long to move the microscope or the preparation, which are both significant masses (considerable inertia). Several technological advances have been introduced in recent years to address this problem. All of them are complex and expensive, typically requiring entirely new systems. In this work, the authors present an elegant solution: move the field of view using mirrors AFTER the objective. This relatively low inertia device enables sub-second sampling of areas in an annulus around the center. The annulus has a diameter much larger than the objective's natural field-of-view.

Although this solution is conceptually simple, there was a considerable amount of engineering that went into it to make the positioning reproducible, as fast as possible, and preserve as much fidelity of the imaging as possible (brightness, numerical aperture). Furthermore, the authors discuss some interesting extensions that are possible with this technology/approach.

Major points:

(1)

There are limitations to this device, and it would be good to expand the discussion of these a bit.

a.

The switching time is relatively slow, limiting frame rates to ~ 5 frames/sec. However, there are plenty of experiments that can be performed adequately using that acquisition speed, as the authors demonstrate.

This is already adequately discussed.

We have added some discussion on the slow switching time (lines 315–323). We have also tested whether this frame rate led to deterioration in the estimation of the pairwise correlation coefficients (CCs) between axonal boutons or between neurons, making comparisons with estimations made using images obtained at a frame rate of 30 Hz (Supplementary Figs. 7 and 8). Pairwise CCs between the inferred spike events of axonal boutons detected at a frame rate of 30 Hz were stably maintained when the imaging data were down-sampled to 5 Hz (correlation coefficient for the CC values between 30 Hz and 5 Hz, 0.91 ± 0.02, *n* = 6 fields), a rate that is similar to the actually used frame rate (4.4 Hz). When the trial-to-trial CCs in the population activity (vectors with time-averaged inferred spike events of neurons imaged during the forelimb movement task) between RFA and CFA were calculated, the CCs were well preserved when the images acquired at 30 Hz were down-sampled to 5 Hz (correlation coefficient for the CC values between 30 Hz and 5 Hz, 0.87 ± 0.02, *n* = 7 fields). Thus, we conclude that the correlation analyses used in the current study are rational, even though the frame rate was relatively slow (~5 Hz).

b.

The NA is also quite low, but it is still usable. This could be discussed a bit more. Maybe one more sentence.

We have added the following sentences to the Discussion section (lines 306–312):
"The axial resolution of approximately 9–12 μm was lower than normally achieved in two-photon microscopy (~2 μm). However, we believe this value to be sufficient to detect calcium transients from single neuronal somata because the soma size is approximately 10 μm and the infrequent occurrence of fluorescence changes (calcium transients) allows us to distinguish different activity patterns from partly overlapping neuronal somata. In addition, when axons or dendrites were sparsely labeled, their calcium transients could be detected (Fig. 6 and Supplementary Fig. 5)"

Also: It is unclear exactly what the NA is. It looks like ~0.28 according to Fig 1e. However, in that caption for that figure, the authors say it is 0.42. In the results section (line 107) it is stated estimated to be 0.36. To users, the precise value isn't so important because, empirically, it works. However, the confusion does a disservice to the paper. Please clear this up.

We are sorry for any confusion regarding the NA. The NA of 0.28 in Fig. 1e in the previous manuscript was the value calculated from simple geometrical optics when 2.5 × 2.5 mm mirrors were used, and the NA of 0.42 in the legend of Fig. 1g in the previous manuscript was the value without the mirrors. The NA of 0.36 mentioned in the previous Results section was the value estimated from the measured axial FWHM of the microbeads. In the revised version of the manuscript, we have directly measured the beam shape passing through the mirrors (Supplementary Fig. 2c, d). The shape was ellipse-like because rectangular mirrors were used (Supplementary Fig. 2b). We also found that the FWHMs of the microbeads were different between the X and Y axes of the FOV and between different rotation angles, depending on the direction of the pair of mirrors (Fig. 2b, d). These results indicate that it is difficult to estimate the practical NA as a single value. Therefore, we agree with the reviewer's comment that the precise NA value is not so important in the current study. In the current manuscript, we have clearly shown the measured lateral and axial FWHMs of the microbeads without estimating the effective NA values. In addition, we have shown the FWHMs of the microbeads measured from the center to the edge of the FOV for five rotation angles and two depths in Fig. 2e–k and Supplementary Figs. 3 and 4.

c.

The access area is limited to an annulus. This limitation isn't adequately discussed. The neurons of interest have to occur somewhere on the annulus, and this can be a significant limitation in some applications.

As pointed out, the imaging area is limited to the donut-shaped area with an outside diameter of ~6.4 mm and inside diameter of ~3.8 mm (5.0–1.2 mm; Fig. 1h). However, by appropriately

moving the center of the donut-shape (i.e., the objective axis) and setting θ, any two FOVs can be sequentially imaged although the edges of the FOVs may sometimes go un-imaged if the distance between the centers of these FOVs is less than the maximum of $d_{m\text{–}m} \times 2 \times \sin(\theta/2)$ (5 mm when $d_{m\text{–}m}$ is 2.5 mm). We have discussed this in the Discussion section (lines 323–330). The maximum length of the rectangular imaging including more than two FOVs was limited to 5.2 mm ($2 \times \sqrt{[3.2^2–1.9^2]}$), the distance between the points at which a tangential line in contact with the inside circumference crosses the outside circumference, and this decreases as the length of the minor side of the rectangle increases. We have added the following sentences to the Results section (line 107–110):

"The maximum observable distance was ~6.4 mm, which is 3.5-fold greater than the diameter of the original FOV, although the donut-shaped center area could not be imaged. The longest length of the line scan with stitched imaging was estimated to be ~5.2 mm (Fig. 1h)."

It is necessary to avoid putting dental cement on the skull in places below which the donut center may be set. We have added a caveat on locating the imaged field within the donut-shaped area to the Methods section (lines 530–539).

To be clear, I am strongly supportive of publication. I am only asking for a slight elaboration on points b and c, and a clarification on point b.

(2)

The authors share most, but not all, of the information necessary to facilitate replication of their device. One omission is the mechanical design. The 3D printing files should be shared, as well as information for the custom machined aluminum case mentioned in the methods. It would also be helpful to see a photograph of the device installed on a microscope as it would be for an experiment.

We have added a structural drawing to Supplementary Fig. 1 and photographs of the device to Fig. 1d–g. We have also added the STL file of the mirror holder for 3D printing to the Supplementary data.

Minor points:

It’s nice to cite colleagues, but it isn’t necessary to compare this work to one photon approaches (ref. 30). If one was to do so, then there would be a lot to add, including SCAPE, lightfield imaging, etc. It simplifies the paper if only multiphoton approaches that work > 200 um deep in the neocortex are compared.

We have removed the comparison between two-photon imaging and one-photon imaging from the current manuscript.

Reviewer #3 (Remarks to the Author):

This manuscript describes an interesting idea and its implementation: using a pair of mirrors positioned after a long-working-distance objective, to rapidly steer the field of view of a 2p microscope to different locations beyond the normal field of view (FOV) of the objective. However, the improvement in FOV, at least as demonstrated, appears to be moderate (6.2 mm vs 5 mm reported), and this improvement is at the expense of greatly reduced NA and poorer axial resolution (12 μm vs 4.1 μm reported) and is associated with a large dead area at the center. Most of biological experiment demonstrations except Figure 3 can be accommodated by existing microscope designs. There also seems to other limitations of the current design (working distance/geometry). Some parameters remained to be reported/tested/discussed.

Specific comments:
1) What is the working distance (after the apparatus)? In figure 1d/e, it indicates that working distance is 1 mm. This is very short. Considering this: for a large glass window 7 mm in size (that is what this microscope is intended for): a 5 degree tilt of the window relative to optical axis would mean 0.6 mm, or most of the working distance. Also, in order to imaging a little bit deeper into tissue, the pair of mirror elements has to nearly directly touch the glass window, which causes many problems, and will be difficult for an average biologist. In the reviewer's experience, even going from a 3 mm to 2 mm working distance (in some available objectives) greatly reduced the usability. Although the element may be moved more upwards closer to the objective to give longer working distance, this maneuver appears to further reduce the NA, which is already very low even at the current design.

We are sorry for the confusion over the definition of the working distance. Once the mirror holder is set between the glass window and the objective, the depth of the focal plane is adjusted only by moving the objective. Therefore, the distance between the bottom surface of the glass window and the bottom surface of the mirror holder is constant ($d_{\text{m–c}}$ in Supplementary Fig. 2a in the current manuscript), and the pair of mirror elements do not directly touch the glass window. When $d_{\text{m–c}}$ was set to be 0.9–1.15 mm, and the mirror height (d_{mh}) and the distance between the mirrors ($d_{\text{m–m}}$) were both 2.5 mm, the objective could be moved up to 1.85–2.1 mm.

This means that, in principle, the imaging depth ranges from 0 to ~2 mm below the cortical surface. In this case, it was not too difficult to perform axial adjustments because the mirror holder was set so that it did not touch the glass window before each imaging session started. Instead, as the reviewer pointed out, the decrease in the distance between the objective and the upper surface of the mirror holder ($d_{o–m}$) decreased the NA because the amount of light passing through the mirrors decreased. We estimated the spatial resolution when the imaging depth was 200, 500, and 800 μm from the cortical surface with $d_{m–c}$ set to 0.9 mm. As shown in Supplementary Fig. 4, the axial FWHMs of the microbeads at the center of the FOV at a depth of 800 μm were only degraded by about 10% compared with the values at a depth of 200 μm. We also estimated the spatial resolution from the center to the edge of the FOV at imaging depths of 200 and 500 μm from the cortical surface ($d_{m–c}$ was set to 1.15 mm). The FWHMs were similar between the two depths (Fig. 2e–k). Thus, for imaging depths limited to 800 μm from the cortical surface, the degradation of the spatial resolution is sufficiently slight to image neuronal somata. We have added these results to the manuscript.

We agree with the reviewer’s comment that the mirror holder and glass window should be parallel as much as possible. This is especially required for imaging through a wide-field cranial window, and we describe its significance in the Methods section (lines 533–539). We have clearly discussed that although the maximum observable distance was ~6.4 mm, the donut center area cannot be imaged (lines 323–330), and that dental cement should not be placed on the donut center (lines 530 and 531).

2) Along the line of the comments above, please provide a few real pictures showing the size, position of the apparatus relative to the microscope and the mouse. This will help the reader to understand the implementation and practicality issues.

We have added a structural drawing to Supplementary Fig. 1 and photographs of the device to Fig. 1d–g. We have also added the STL file of the mirror holder for 3D printing as Supplementary data.

3) What is the resolution/NA at the edge of FOV? While for all objectives, the performance at edge is

poorer than center, the reviewer worries that the need to pass beam through the small aperture may further reduced NA at the edge with the current design, and the resolution/light collection may be poor. These factors may reduce the practical FOV.

We measured the FWHMs of the microbeads from the center to the edge of the FOV. The maximum degradation from the center to the edge (500 μm from the center) was ~20% (Fig. 2e–g). The fluorescent intensity at the edge of the FOV was ~30% weaker than at the center (Fig. 2h). Thus, calcium transients can be detected from single neuronal somata throughout the 1000 μm × 1000 μm area of the FOVs, even though the number of detected neurons at the periphery of the FOV might be smaller than at the center.

4) The area at the center that cannot be imaged is quite big, occupying ~40-60% of the area depending on the practical FOV at a given position. How does this affect experimental designs? What experimental limitations come with this property? Some discussions on this topic are needed.

As the reviewer pointed out, the imaging area is limited to the donut-shaped area with an outside diameter of ~6.4 mm and inside diameter of ~3.8 mm (5.0–1.2 mm; Fig. 1h). However, by appropriately moving the center of the donut-shape (i.e., the objective axis) and setting θ, any two FOVs can be sequentially imaged although the edges of the FOVs may sometimes go un-imaged if the distance between the centers of these FOVs is less than the maximum of $d_{m–m} \times 2 \times \sin(\theta/2)$ (5 mm when $d_{m–m}$ is 2.5 mm). The maximum length of the continuous line imaging including more than two FOVs was limited to 5.2 mm, the distance between the points at which a tangential line in contact with the inside circumference crosses the outside circumference, and this decreases as the length of the short side of the rectangle increases. We have added the following sentences to the Results section (line 107–110):
"The maximum observable distance was ~6.4 mm, which is 3.5-fold greater than the diameter of the original FOV, although the donut-shaped center area could not be imaged. The longest length of the line scan with stitched imaging was estimated to be ~5.2 mm (Fig. 1h)."

It is necessary to avoid putting dental cement on the skull in places below which the donut center may be set. We have added a caveat on locating the imaged field within the donut-shaped area to the Methods section (lines 530–539).

5) The use of an aperture that is not round/square, as shown in figure 1g, means that the NAs for x and y are different. How does this affect the resolution and/or the point spread function? Some discussions would be nice.

We directly measured the shape of the laser beam with and without the mirrors. As expected, the laser beam passing through the rectangular mirrors was ellipse-like (Supplementary Fig. 2d). When we measured the lateral FHWMs of the microbeads along the major and minor axes of the mirrors, the lateral FWHM along the major axis was 1.26 μm, which was 1.43-fold longer than that along the minor axis (0.88 μm). This was consistent with the ratio of the major axis length to the minor axis length (4.0/2.5 = 1.6). We have added this result to Supplementary Fig. 2. We have also shown FWHMs along two lateral axes at five rotation angles (Fig. 2b–d). The mean of the lateral FWHMs along the major and minor axes was similar across any angle, indicating that the rectangular mirror shape does not have large effects on the imaging of neuronal soma with a diameter of 10–15 μm. We have added these results and a discussion in lines 119–131.

6) Figures 1f and 1g are confusing. The authors show an “inferred” beam size. Can this be replaced by a directly measurement? Is there an explanation that the beam shape is square? Also, by using a rectangular aperture as large as the beam area, when the aperture is rotated to a different angle, would part of the aperture not covered by the beam (and this would decrease NA)?

We are sorry for this confusion. As described above, we directly measured the beam shape passing through the mirrors. The shape was ellipse-like because rectangular mirrors were used (Supplementary Fig. 2b, d). The FWHMs of the microbeads differed between the X and Y axes, depending on the mirror rotation angle (Fig. 2b). These results indicate that the lateral spatial resolution depended on the direction of the pair of mirrors. We also measured FWHMs from the

center to the edge of the FOV for five rotation angles and two depths. As reviewer 2 pointed out, the precise NA value is not so important in this study. We have clearly shown the measured lateral and axial FWHMs of the microbeads in Fig. 2 and Supplementary Figs. 3 and 4, without estimating the effective NA values.

Reviewer #4 (Remarks to the Author):

Comments about the biology in the submission "Super-wide-field two-photon imaging with a micro-optical device 2 moving in post-objective space".

The authors utilize their new wide field imaging approach in attempts to learn something about inter-area connections and correlations. They attempt to use their microscope and correlation in calcium activity to trace single axons in vivo. While I think this is an interesting approach, the authors fail to convince me that they actually accomplished this goal. As a matter of fact, the only conclusion they make is "probably represented the same neuron". No alternative evidence is given that they did trace an axon. Since this method is based on correlations in activity, the slow imaging speed is also a significant problem, leading to potential false positives.

To test whether the pairwise correlation coefficients (CCs) between axonal boutons were reliable in the images acquired at 4.4 Hz, we conducted two-photon calcium imaging of CFA L1 axons projecting from the RFA at 30 Hz. Then, we down-sampled the imaging data to 15, 5, 3, 2, 1, 0.5, and 0.25 Hz, and calculated the pairwise CCs between the detected axonal boutons for each sampling rate (Supplementary Fig. 7). The correlation coefficient for the pairwise CCs between 30 Hz and 5 Hz data was 0.91, and between 30 Hz and 3 Hz it was 0.89 (Supplementary Fig.7d, e). These high values were probably helped by the slow kinetics of GCaMP6s (~1 s decay time constant), and the fact that the activity in each neuron was relatively sparse. These results suggest that when activity is sparse and synchronous activity in the same axon is only rarely contaminated by the spontaneous activity of other axons occurring in close proximity, the probability of including false positive axonal boutons in a cluster of highly-correlated axonal boutons is small, even at the frame rate of 4.4 Hz. We have also described this prerequisite in lines 231–234. Next, we examined whether a given RFA L5 neuron could show highly-correlated activity with multiple CFA L1 axonal boutons by chance. For each neuron in the RFA L5 field shown in Fig. 6c, we calculated the pairwise CCs against individual axonal boutons in the CFA L1 field and sorted them in order from the highest value for each neuron (Fig. 6f). We found that only one RFA neuron showed CCs of more than 0.6 with

multiple axonal boutons in the CFA. This neuron had much lower CCs with all other axonal boutons, and the highest CCs corresponded to 6.5 s.d. of the CC distribution (Fig. 6g, red arrowhead). This suggests that the false positive rate was very small and that no neurons had high CCs with multiple boutons by chance. We have added these down-sampling experiments to Supplementary Fig. 8, and the result regarding the distribution of the pairwise correlations to Fig. 6.

The other use case the authors attempted was looking at dynamics between cortical areas with cellular and subcellular resolution. Again, this is important but I do not think we learned much new from this approach. Interarea comparisons have already been made between cortical areas without cellular resolution. Here, the cellular resolution is used to look at correlations between neurons, but again the very slow imaging speeds limits the conclusions about observed correlations. Controls for other differences between areas also need to be performed, such as differences in somata depth, differences in expression level, differences in calcium buffering, and differences in action potential sensitivity. Comparisons between RFA and CFA have been done by the labs of Whishaw, Murphy, and He, all of which are not cited in this manuscript. This work needs to be put in context with the literature.

In the current version of the manuscript, we consider whether trial-to-trial neuronal population activities in the RFA and CFA are mutually related or independent. This is because it has not been solved although the RFA and CFA are strongly related to forelimb movement and coordinately activated during performance of forelimb movement tasks, as the reviewer pointed out (Farr & Whishaw, Stroke 33, 1869-1875, 2002; Harrison et al., Neuron 74, 397-409, 2012; Hira et al., J Neurosci. 33, 1377-1390, 2013; Makino et al., Neuron 94, 880-890, 2017; Kimura et al., J Physiol. 595, 385–413, 2017; Wang et al., Cell 171, 440-455, 2017). To simplify the matter and increase the frame rate, we sequentially imaged RFA L2/3 and CFA L2/3 at 5.4 Hz while mice performed a sound-cue-triggered lever-pull task. Because of this relatively slow imaging rate, we did not examine the time course of the inferred spike events in each trial, but instead calculated the time-averaged inferred spike events (from 0.46 s before to 1.85 s after the onset of each successful lever pull) of task-related neurons, and defined a vector with the time-averaged activity of task-related neurons for each trial as the population activity. Then, we

estimated the trial-to-trial correlation in the population activity. The population activity showed low trial-to-trial CCs (Fig. 7e, f; 0.10 ± 0.018 in RFA L2/3 and 0.12 ± 0.026 in CFA L2/3, n = 11; please see Methods for details of the analytical procedures), even though the field-averaged fluorescence change and lever trajectory were relatively stable across successful lever-pull trials (Fig. 7f; trial-to-trial correlation coefficient of field-averaged fluorescence change: 0.95 ± 0.01 in RFA L2/3 and 0.87 ± 0.02 in CFA L2/3; trial-to-trial correlation coefficient of lever trajectory, 0.89 ± 0.03, n = 11). This suggests that the population activity in the high dimensional space formed by multiple neurons differed considerably across trials. Next, we examined whether the trial-to-trial population activity was similar between the RFA and CFA. The successful trials for each imaging session were classified into 8–14 clusters according to the similarity of the RFA population activity within the successful trials (RFA-dependent cluster; Fig. 8a–c; please see Methods for details). The correlation in the CFA population activity within the clusters was higher than that between randomly chosen trials (Fig. 8c, d). We obtained similar results when the cluster was determined from the similarity of the CFA population activity within the successful trials (CFA-dependent cluster) and the RFA population activity within the clusters was examined (Fig. 8d). Calculation of these correlations from the imaging data acquired at a frame rate of 5.4 Hz was rational because the trial-to-trial correlation coefficient was well preserved when the images acquired at 30 Hz were down-sampled to 5 Hz (Supplementary Fig. 8). The distances of the population activity in the RFA between different clusters correlated strongly with that of the CFA between the different clusters (Fig. 8e–g). The cluster classification was not simply related to the similarity of the lever-pull speed, lever trajectory, reaction time, or reward history within each cluster (Supplementary Fig. 9a, b; please see Methods for details). Even in a small subset of the clusters with high similarity for these properties, the correlation in these properties within each cluster was not different from that between different clusters (Supplementary Fig. 9c, d). In addition, trial clusters determined by the population activity were not simply related to the similarity in the field-averaged fluorescence change within each cluster (Supplementary Fig. 9e–h). These results suggest that trial-to-trial population activity in the RFA and CFA is mutually related, and that it was not simply because of the trial-to-trial similarity in behaviors, or the net activity between the RFA and CFA, but was probably a result of the network dynamics embedded in the RFA and CFA. The novelty of these finding lies in the fact

that they cannot be obtained from analysis of field-averaged activity, and thereby demonstrate the suitability of the super-wide-field TLPSM for performing correlation analyses of neuronal populations. We have added these results and statistics to the last subsection of the Results section, and to Fig. 8 and Supplementary Figs. 8 and 9.

Although we took care to balance the injections, we found a small but significant difference in the decay time constants of the calcium transients between RFA and CFA neurons. This difference could possibly cause differences in the inference of the neuronal activity. However, the mean, standard deviation, and maximum values of the inferred spike events during the non-lever moving periods in each field were not significantly different between RFA and CFA neurons. Within the range of our experiment, the mean CC in the population activity between pairs of trials did not correlate with the decay time constants, or with the number of neurons detected in each field. Thus, we concluded that the differences in the fluorescence expression between the RFA and CFA neurons did not have large effects on the results regarding the correlation analyses. We have added these results, with the statistics, to the Methods section (lines 653–670).

In this analysis, we did not use the time course of activity during movement, which requires measurements with a high temporal resolution. Clarification of the dynamics of time-evolving population activity is critical for understanding the motor control system (Churchland et al., Nature 487, 51–56, 2012). We have discussed the limitation of our current experiment in the Discussion section (lines 351–360).

Reviewers' comments:

Reviewer #1 (Remarks to the Author):

The authors addressed all my concerns. I recommend publication.

Reviewer #2 (Remarks to the Author):

I am pleased with this revision, and I support publication. Nice work.

Reviewer #3 (Remarks to the Author):

The authors have addressed my specific concerns although the general comments (moderately improved FOV compared to existing designs, greatly reduced NA and poor axial resolution, and large dead center) remained. However, given its potential to become a relatively low-cost add-on to existing microscopes, the current method may still see certain applications.

I have a few additional comments below:
1) Supplementary Figure 2a is not accurate, as it does not reflect the additional focusing depths.
2) Using a NA matched objective, instead of the 0.8NA objective may provide equal performance, while having lower cost and/or longer working distance. This point may be worth of discussing.

Reviewer #4 (Remarks to the Author):

In the manuscript, "Super-wide-field two-photon imaging with a micro-optical device moving in post-objective space" the authors compare trial to trial variability across RFA and CFA. Most of these comparisons are based on inferred spikes. However it is unclear how error prone this analysis is. What is the false positive and false negative rates of inferred spikes? Could these errors be responsible for high trial to trial variability? Also, the authors should more conclusively show that the behavior is not responsible for the variability. For example if one tries to cluster trials based upon the features of the movement and trial types, would similar clusters emerge. Until this is resolved it is unclear if the variability is coming from the behavior or reciprocal connectivity between RFA and CFA.

The authors demonstrate that their slow imaging speeds are fine because if they downsample 30Hz imaging data they get the same result. The argument could be made that this is because 30Hz imaging is also inappropriate for studies of motor control.

We thank the reviewers for their careful consideration of our manuscript and helpful comments. Our detailed responses to the reviewers' comments are provided below:

Reviewers' comments:

Reviewer #3 (Remarks to the Author):

The authors have addressed my specific concerns although the general comments (moderately improved FOV compared to existing designs, greatly reduced NA and poor axial resolution, and large dead center) remained. However, given its potential to become a relatively low-cost add-on to existing microscopes, the current method may still see certain applications.

I have a few additional comments below:
1) Supplementary Figure 2a is not accurate, as it does not reflect the additional focusing depths.

We have added "z", the distance between the bottom surface of the coverslip and the focal plane (corresponding to the focusing depth under the cortical surface *in vivo*).

2) Using a NA matched objective, instead of the 0.8NA objective may provide equal performance, while having lower cost and/or longer working distance. This point may be worth of discussing.

We have added the following sentences in the Discussion section (lines 317–320):
"If the present spatial resolution is sufficient, the NA of the objective could be reduced to match the mirror area (from 0.6 to ~0.4). Low NA objectives generally have long WDs, which may allow us to increase the distance between the centers of the two FOVs, $d_{m\text{–}m} \times 2 \times \sin(\theta/2)$."

Reviewer #4 (Remarks to the Author):

In the manuscript, “Super-wide-field two-photon imaging with a micro-optical device moving in post-objective space” the authors compare trial to trial variability across RFA and CFA. Most of these comparisons are based on inferred spikes. However it is unclear how error prone this analysis is. What is the false positive and false negative rates of inferred spikes? Could these errors be responsible for high trial to trial variability? Also, the authors should more conclusively show that the behavior is not responsible for the variability. For example if one tries to cluster trials based upon the features of the movement and trial types, would similar clusters emerge. Until this is resolved it is unclear if the variability is coming from the behavior or reciprocal connectivity between RFA and CFA.

We thank the reviewer for the careful reviewing and helpful comments on the analysis of trial-to-trial variability in the population activity. The GCaMP6s used in the current study is highly sensitive to a single spike event, so much so that it has been reported to detect 99% of single spikes at a 1% false-positive rate (Chen et al., Nature 499, 295–300, 2013). Since multiple firing events frequently occur in a time bin at the frame rate for calcium imaging (i.e., 30 Hz), the correlation coefficient between the inferred and the original spike rates, rather than false-positive and false-negative rates, is used as a performance index for the spike inference algorithm (Smith and Häusser, Nat Neurosci 13,1144–1149, 2010; Theis et al., Neuron 90, 471–482, 2016). Thesis et al. (2016) reported that, in simultaneous in vivo calcium imaging and cell-attached recording of L2/3 GCaMP6s-expressing neurons in the mouse V1, the correlation coefficient is 0.3~0.5 when the time bin was 50 ms and ~0.8 when the time bin was 500 ms. As the CNMF algorithm used in the current study is relatively robust to the noise level of the fluorescence signals (Pnevmatikakis et al., Neuron 89, 285–299, 2016), we assume that these spike inference properties basically held true in our current study. Therefore, we consider that the trial-averaged activity with a time bin of ~2.3 s used in the correlation analysis (Figure 8) reflected well the summed true spike rates during this time bin. However, without simultaneous electrical recording, it was difficult to accurately estimate the false-positive and false-negative rates of inferred spike events or the correlation between the inferred and the original spike rates in each mouse in our study. Instead, to remove the effect of spike inference errors on the

correlation analysis, we used non-deconvolved Δ*F/F* traces as the RFA and CFA population activity. When non-deconvolved Δ*F/F* traces were used for trial clustering, the intra-cluster correlation coefficient (CC) in the CFA population activity from the RFA-dependent clusters (0.26 ± 0.02, n = 11) was significantly higher than that from shuffled data (0.06 ± 0.01; $p = 5.3 \times 10^{-6}$, paired *t*-test) and the intra-cluster CC in the RFA population from the CFA-dependent clusters (0.27 ± 0.03, n = 11) was also significantly higher than that from shuffled data (0.10 ± 0.02; $p = 8.5 \times 10^{-5}$, paired *t*-test) (Response Fig. 1a). The correlation coefficient for the regression line between cluster distance in the RFA and CFA was 0.57 ± 0.05 (n = 11) for the RFA-dependent cluster and 0.48 ± 0.08 for the CFA-dependent cluster (Response Fig. 1b). These values were similar to those obtained when inferred spike events were used (Fig. 8g). Thus, we concluded that the results shown in Figure 8 were not caused by errors that might occur during the spike inference procedure. We have added the statistical results to the Methods section (lines 712–721).

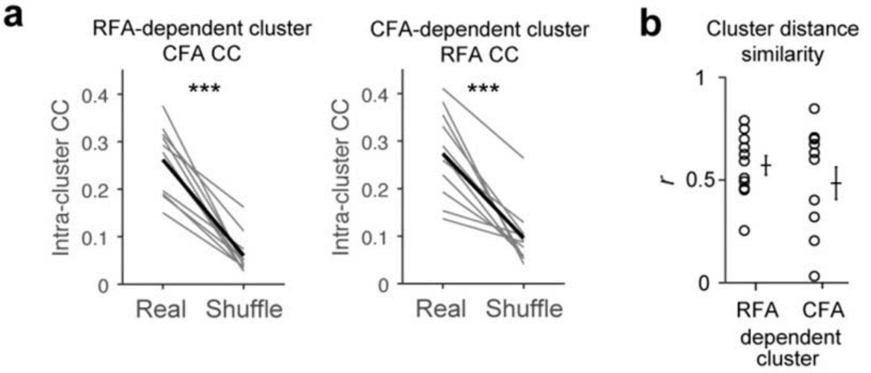

Response Figure 1 | Relationships in trial-to-trial population activity between RFA and CFA when non-deconvolved Δ*F/F* traces were used.

(**a**) Averaged trial-to-trial population activity CC within the clusters (intra-cluster CC). In each field, the CCs between pairs of trials within each cluster were averaged, and these values were averaged across clusters. Shuffled intra-cluster CCs were calculated by shuffling the trials. Gray lines indicate individual pairs and black lines mean values. ***: $p < 0.001$, paired *t*-test (n = 11).
(**b**) The correlation coefficient (r) for the regression line between cluster distance in the RFA and CFA in each imaging session (n = 11). The distance was defined as the correlation coefficient in

the averaged population activity between clusters. Crosses indicate mean ± s.e.m. The left and right sides are the results from the RFA-dependent and CFA-dependent clusters, respectively.

We examined the intra-cluster similarity in the population activity when the trial cluster was determined according to the similarity of the four behavioral properties (behavior-dependent cluster). The intra-cluster CCs in the CFA population activity and the intra-cluster CCs in the RFA population activity from the behavior-dependent cluster were higher than those from shuffled data (Supplementary Fig. 9f). However, the intra-cluster CCs in the CFA population activity from the behavior-dependent cluster were lower than those from the RFA-dependent cluster, and the intra-cluster CCs in the RFA population activity from the behavior-dependent cluster were lower than those from the CFA-dependent cluster (Supplementary Fig. 9f). We have added these results to Supplementary Fig. 9e, f. To remove the effect of the difference between the number of the behavior-dependent cluster per imaging session (7.6 ± 0.6, n = 11) and those of the population activity-dependent clusters (RFA-dependent, 10.4 ± 0.6; CFA-dependent, 10.0 ± 0.7), we reset the 75th percentile of the elements of the distance matrix for the affinity propagation calculation to obtain a similar number of clusters (10.9 ± 0.5). In this case, the result was similar (Response Figure 2). Furthermore, to remove a possibility that one or two behavioral properties were not related to the population activity at all and a distractor for clustering, we also clustered the trials based on two or three of the four behavioral properties. However, this was not the case because these intra-cluster CCs were lower than when the four properties were used for clustering. These results suggest that the behavioral similarity was less related to the similarity of the RFA (or CFA) population activity within trial clusters than the similarity of the CFA (or RFA) population activity was. We have referred to this in the conclusion in the Discussion section (lines 353–356). Thus, we do not conclude that the behaviors were not responsible for the variability. The four behavioral properties might be insufficient to cluster the trials with similar population activity. We mention the future necessity to introduce high-speed videography of orofacial behavior and postures in the Discussion section (lines 367–370).

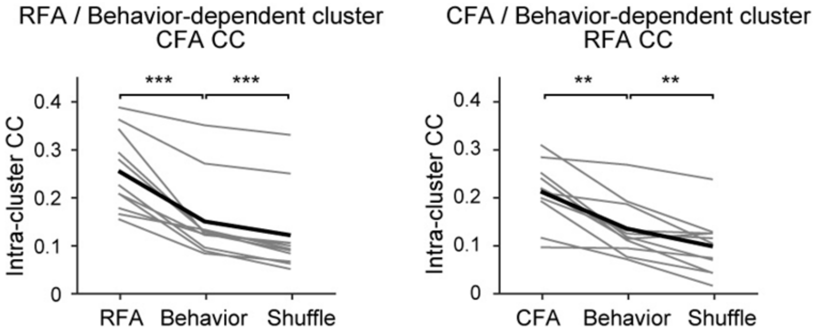

Response Figure 2 | Intra-cluster CCs in the population activity from population activity-dependent and behavior-dependent clusters.

Left, field-averaged intra-cluster CCs in the CFA population activity from the RFA-dependent cluster, behavior-dependent cluster, and shuffled data based on the behavior-dependent cluster. In this analysis, the behavior-dependent cluster was determined by the affinity propagation with the 75th percentile, not the median, of the elements of the distance matrix. Right, field-averaged intra-cluster CCs in the RFA population activity from the CFA-dependent cluster, behavior-dependent cluster, and shuffled data based on the behavior-dependent cluster. ***: $p < 0.001$, **: $p < 0.01$, multiple paired *t*-test with Bonferroni correction ($n = 11$).

The authors demonstrate that their slow imaging speeds are fine because if they downsample 30Hz imaging data they get the same result. The argument could be made that this is because 30Hz imaging is also inappropriate for studies of motor control.

As the reviewer pointed out, it is difficult for two-photon calcium imaging at a frame rate of ≤ 30 Hz to detect spike synchronization and γ oscillation, such as those observed in the motor cortex (Riehle et al., Science 278, 1950–1953, 1997; Hatsopoulos et al., Proc Natl Acad Sci USA 95, 15706–15711, 1998; Igarashi et al., J Neurosci 33, 18515–18530, 2013). We have added this limitation (lines 364–366).
